# Improving anti-cancer drug delivery performance of magnetic mesoporous silica nanocarriers for more efficient colorectal cancer therapy

**DOI:** 10.1186/s12951-021-01056-3

**Published:** 2021-10-12

**Authors:** Sonia Iranpour, Ahmad Reza Bahrami, Sirous Nekooei, Amir Sh. Saljooghi, Maryam M. Matin

**Affiliations:** 1 Department of Biology, Faculty of Science, Ferdowsi University of Mashhad, Mashhad, Iran; 2 Industrial Biotechnology Research Group, Institute of Biotechnology, Ferdowsi University of Mashhad, Mashhad, Iran; 3 Department of Radiology, Faculty of Medicine, Mashhad University of Medical Sciences, Mashhad, Iran; 4 Department of Chemistry, Faculty of Science, Ferdowsi University of Mashhad, Mashhad, Iran; 5 Novel Diagnostics and Therapeutics Research Group, Institute of Biotechnology, Ferdowsi University of Mashhad, Mashhad, Iran

**Keywords:** Colorectal cancer, Drug delivery system, pH-sensitive gatekeeper, Targeted therapy, Theranostic, Magnetic mesoporous silica NPs

## Abstract

**Background:**

Improving anti-cancer drug delivery performance can be achieved through designing smart and targeted drug delivery systems (DDSs). For this aim, it is important to evaluate overexpressed biomarkers in the tumor microenvironment (TME) for optimizing DDSs.

**Materials and methods:**

Herein, we designed a novel DDS based on magnetic mesoporous silica core–shell nanoparticles (SPION@MSNs) in which release of doxorubicin (DOX) at the physiologic pH was blocked with gold gatekeepers. In this platform, we conjugated heterofunctional polyethylene glycol (PEG) onto the outer surface of nanocarriers to increase their biocompatibility. At the final stage, an epithelial cell adhesion molecule (EpCAM) aptamer as an active targeting moiety was covalently attached (Apt-PEG-Au@NPs-DOX) for selective drug delivery to colorectal cancer (CRC) cells. The physicochemical properties of non-targeted and targeted nanocarriers were fully characterized. The anti-cancer activity, cellular internalization, and then the cell death mechanism of prepared nanocarriers were determined and compared in vitro. Finally, tumor inhibitory effects, biodistribution and possible side effects of the nanocarriers were evaluated in immunocompromised C57BL/6 mice bearing human HT-29 tumors.

**Results:**

Nanocarriers were successfully synthesized with a mean final size diameter of 58.22 ± 8.54 nm. Higher cytotoxicity and cellular uptake of targeted nanocarriers were shown in the EpCAM-positive HT-29 cells as compared to the EpCAM-negative CHO cells, indicating the efficacy of aptamer as a targeting agent. In vivo results in a humanized mouse model showed that targeted nanocarriers could effectively increase DOX accumulation in the tumor site, inhibit tumor growth, and reduce the adverse side effects.

**Conclusion:**

These results suggest that corporation of a magnetic core, gold gatekeeper, PEG and aptamer can strongly improve drug delivery performance and provide a theranostic DDS for efficient CRC therapy.

**Graphic abstract:**

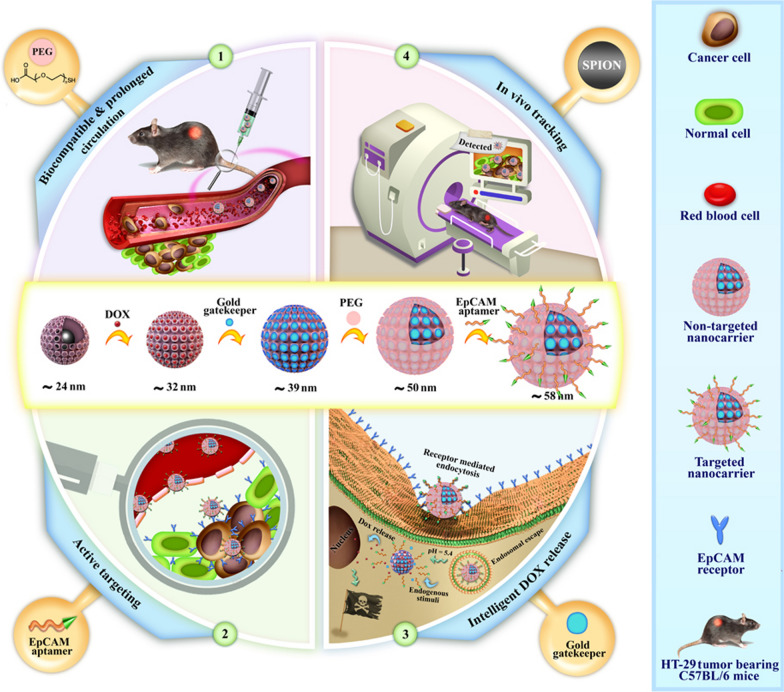

**Supplementary Information:**

The online version contains supplementary material available at 10.1186/s12951-021-01056-3.

## Background

Cancer is one of the most devastating problems in the world, and colorectal cancer (CRC) has been reported as the second leading cause of cancer related death in both sexes as evident from the GLOBOCAN database [1]. In this regard, researchers are constantly searching for novel and more effective treatment strategies to improve therapeutic outcomes with focus on patients’ convenience. Nanotechnology has had a great impact on cancer treatment field by introducing various types of theranostic nano-delivery platforms [2]. Incorporation of traceable imaging modalities and therapeutic compounds on a single nano-delivery system provides a theranostic platform which results in efficient targeted therapy through image-guided drug delivery [3]. Generally, in order to improve drawbacks of conventional anti-cancer drugs and maximize drug delivery performance, it is necessary to consider two crucial aspects including (1) physicochemical parameters of nanoparticles (NPs) and (2) tumor microenvironment (TME) features.

Mesoporous silica nanoparticles (MSNs) constitute one of the most important classes of inorganic nanomaterials which can be used as an effective drug delivery system (DDS)/or nanocarrier [4]. MSN has received much attention due to its unique properties including porosity [5], dual/or multiple drug loading capacity [6, 7], biodegradable characteristics [8], biocompatible features [9], controlled drug release and incorporation with detectable agents [10, 11]. Intelligent drug release from MSNs at the intended site can be managed by conjugation of specific gatekeepers at pore entrances which can be controlled by endogenous and/or exogenous stimuli [12]. Generally, the pH value of TME is slightly lower than normal tissues [13]; therefor functionalized MSNs with pH-responsive gatekeepers such as gold NPs can effectively turn closed tunnels to an open state, in response to an endogenous stimulus, and regulate the release of guest molecules [14]. In this context, several smart pH-responsive MSNs based on gold NPs have been developed for an on-demand release of anti-cancer drugs [15, 16]. Moreover, some critical challenges such as half-life of nanocarriers in the blood, their toxicity, hemolysis of erythrocytes, and recognition of DDSs by host immune cells need to be addressed prior to the design, synthesis, and widespread employment of DDSs. It has been proven that PEGylation method is a mainstay strategy to overcome some of the mentioned problems [17, 18]. Polyethylene glycol (PEG), an FDA-approved polymer for different drugs [19], can effectively cover the MSN surface and make it favorable for systemic administration, resulting in improved pharmacological properties of nanocarriers and optimizing the drug delivery behavior [20].

Generally, nanocarriers can passively reach the tumor site through enhanced permeability and retention (EPR) effect [21], and specific recognition of cancer cells in the TME is an important step to exert their destructive effects. Therefore, screening and identification of particular overexpressed cancer cell receptors in the TME is an effective way for selective delivery of therapeutic drugs. This approach is called targeted drug delivery and specific moieties such as various aptamers have been designed and conjugated on the surface of DDSs to improve identifying their cognate receptors on cancer cells. Targeted drug delivery strategy maximizes availability of anti-cancer drugs at the tumor site, significantly minimizes adverse side effects, and ultimately helps to improve clinical outcomes [22, 23]. Epithelial cell adhesion molecule (EpCAM; CD326) is a cell surface receptor which is abundantly overexpressed in the rectal cancer tissue as compared with adjacent normal tissues [24], so it can be considered as an ideal CRC biomarker in active targeting. In this regard, Xie et al. increased the therapeutic efficacy of DOX against SW620 colon cancer cells by modification of MSNs with a DNA EpCAM aptamer and showed considerably increased toxicity of DOX in comparison with non-targeted MSNs [25]. Another group designed multifunctional MSNs with focusing on intelligent drug release, high circulatory half-life, and selective delivery of a maytansine derivative (DM1) for CRC treatment. For this aim, hydrochloride polydopamine (PDA) as a well-known gatekeeper, PEG and DNA EpCAM aptamer were applied and created a favorable platform for CRC therapy. Both in vitro and in vivo results confirmed advantages of prepared MSNs in specific targeting of cancer cells with low off-target toxicity [26]. Moreover, Gao et al*.* applied two aptamers on the surface of MSNs in order to improve specific recognition of colon cancer cells, overcome the heterogeneity patterns and prevent lung metastasis. They showed modification of MSNs with both EpCAM and CD44 aptamers could coordinately restrain proliferation and metastasis of SW620 colon cancer cells [27].

Herein, we improved anti-cancer drug delivery performance by designing smart and targeted magnetic mesoporous silica core–shell NPs as a theranostic nano-delivery platform for CRC therapy. For this aim, superparamagnetic iron oxide nanoparticles (SPIONs) as magnetic core were conjugated with MSNs to make a theranostic platform for in vivo monitoring of therapeutic response. Subsequently, DOX was encapsulated into the tunnels and its intelligent release in the tumor cells was facilitated via introducing pH-responsive gold gatekeepers at the surface of MSNs. In the final step, a bi-functional PEG or DNA EpCAM aptamer (SYL3C; introduced by Song et al*.* [28]) was attached to prepared non-targeted and targeted DDSs, respectively. Incorporation of magnetic core for MRI imaging purposes, smart drug release at acidic pH, and specific cancer cell targeting are three important considerations of this work. After fully characterization of prepared nanocarriers, the anti-cancer activity, cellular internalization, and cell death mechanism of non-targeted and targeted nanocarriers were compared in vitro. Finally, tumor inhibitory effects, biodistribution and possible side effects of the nanocarriers were evaluated in immunocompromised C57BL/6 mice bearing human HT-29 tumors.

## Results

### Characterization

**Fig. 1:**
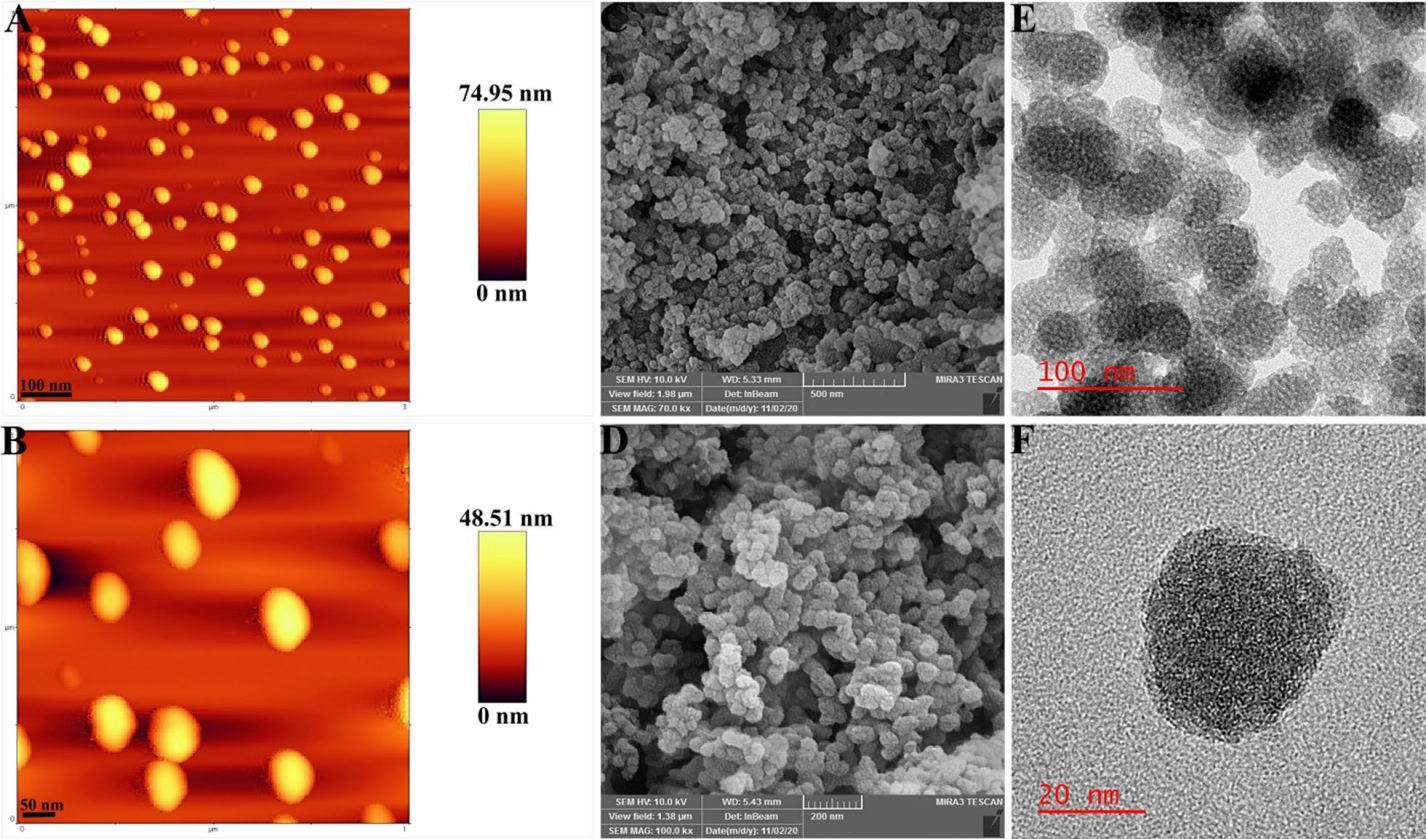
Morphological characterizations of the backbone. **A**, **B** AFM, **C**, **D** SEM and **E**, **F** HR-TEM images of SPION@MSNs. *AFM* Atomic force microscopy, *SEM* Scanning electron microscopy, *HR-TEM* High resolution-transmission electron microscopy, *SPION* Superparamagnetic iron oxide nanoparticle, *MSN* Mesoporous silica nanoparticle

**Fig. 2:**
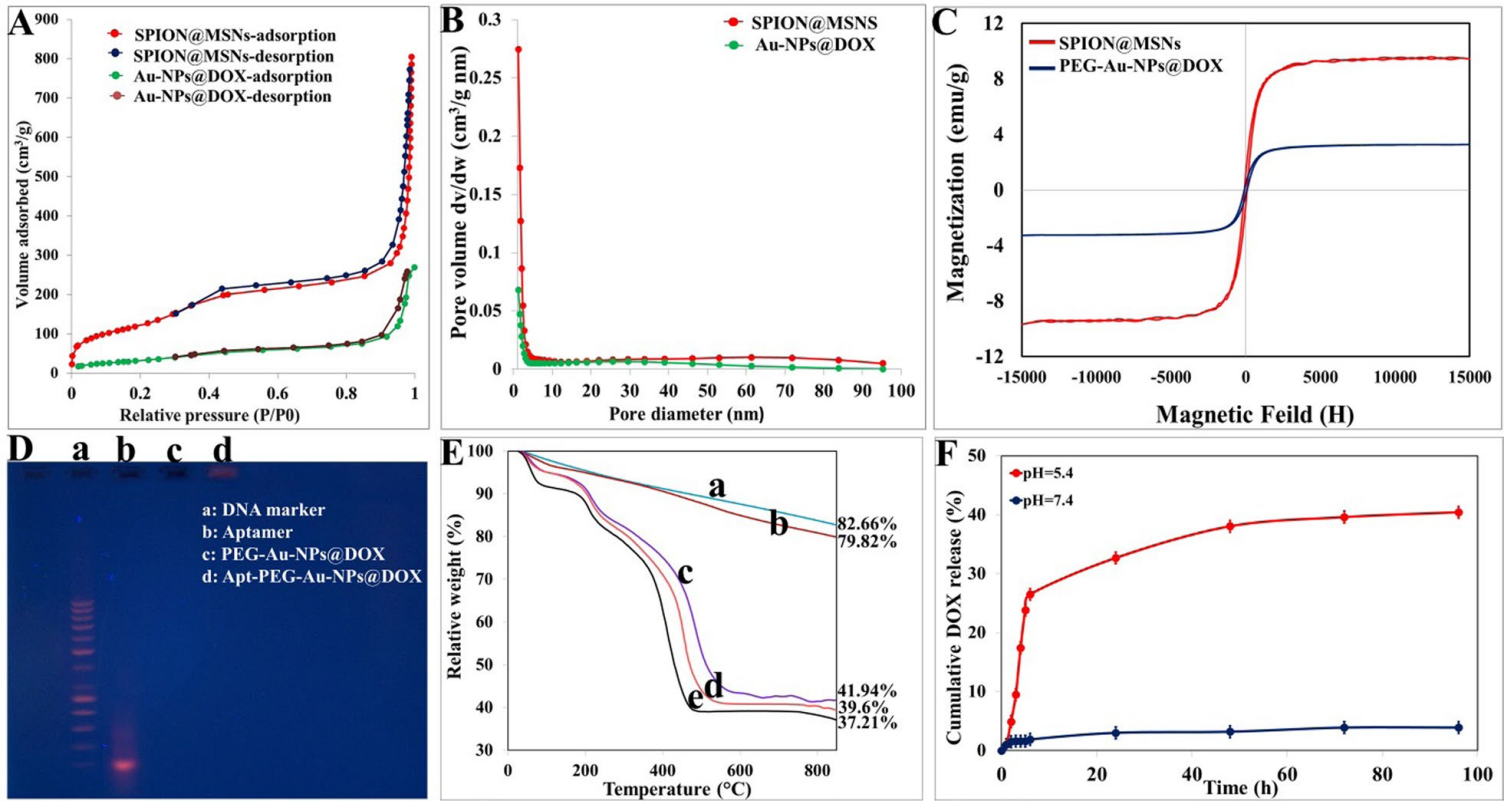
Physicochemical characterization of nanocarriers. **A** N_2_ adsorption/desorption isotherms and **B** pore size distribution of SPION@MSNs and Au-NPs@DOX. **C** VSM curves of SPION@MSNs and PEG-Au-NPs@DOX. **D** The conjugation of EpCAM aptamer was verified by electrophoresis. **E** TGA analysis curves of (a) SPION@MSNs, (b) SPION@MSNs-NH_2_, (c) Au-NPs@DOX, (d) PEG-Au-NPs@DOX and (e) Apt-PEG-Au-NPs@DOX. **F** In vitro DOX release profile of Au-NPs@DOX at pH 7.4 and pH 5.4 (data presented as mean ± SD, n = 3). *SPION* Superparamagnetic iron oxide nanoparticle, *MSN* Mesoporous silica nanoparticle, *NP* nanoparticle, *DOX* Doxorubicin, *VSM* Vibration sample magnetometer, *PEG* Polyethylene glycol, *Apt* Aptamer, *EpCAM* Epithelial cell adhesion molecule, *TGA* Thermal gravimetric analysis

In present study the main backbone, SPION@MSN, was first prepared and fully characterized. The FTIR spectra (Additional file 1: Figure S1) indicated the bands at 572 cm^−1^ corresponding to the Fe–O bond, which was covered by the absorption bands of Si–O-Si and Si–O at 1090 and 632 cm^−1^, respectively [29]. Moreover, AFM and SEM results demonstrated that nanocarriers had a uniform spherical morphology (Fig. 1A–D) and TEM images showed that SPION@MSNs had a mean diameter of ~ 20 nm with an open porous structure throughout the entire MSNs (Fig. 1E, F). As shown in Table 1, the mean zeta potential and particle size of SPION@MSNs were determined around − 19.52 ± 1.85 mV and 24.37 ± 0.21 nm, respectively. To prove the porous nature of the backbone, N_2_ absorption/desorption isotherms was performed and showed a typical type IV isotherm along with type-H1 hysteresis loop, giving a large surface area (432.79 m^2^/g), pore volume (1.23 cm^3^/g), and thin pore size 1.21 nm (Fig. 2A, B). Eventually, the magnetization curve demonstrated that the saturated magnetization value of SPION@MSNs was about 9.75 emu/g (Fig. 2C). After modification of core–shell surface with amine groups, the two bands at 3000 and 1556 cm^−1^ in the FTIR spectrum were emerged and assigned to N–H and C=N groups, respectively [30]. Moreover, presence of N element in the prepared formula was further confirmed by EDX elemental analysis (Additional file 1: Figure S2). The surface charge increased to + 15.35 ± 3.23 mV, which was attributed to greatly abundant N_2_ groups strongly attached on the MSN surface and the average size observed in DLS was around 27.44 ± 1.83 nm. DOX loading in the porous cavity was performed by stirring DOX and SPION@MSNs-NH_2_ continuously at a w/w ratio of 1:1. The encapsulation efficiency (EE%) and drug loading capacity (LC%) were about 98.65% ± 0.88 and 49.79% ± 1.03, respectively according to the data obtained by UV/Vis spectroscopy. In the FTIR spectra, absorption peaks at 3436 and 1728 cm^−1^ were observed due to additional hydroxyl and carbonyl groups, respectively [31] verifying the effective loading of DOX on the silica pores. The zeta potential and particle size were recorded around − 10.55 ± 1.00 mV and 7.92 ± 3.33 nm, respectively for NPs@DOX formula. In the next step, gold NPs as pH responsive gatekeepers were synthesized to cap the pore entrances of MSNs. The obtained gold gatekeepers were characterized on the basis of morphology, functional groups, maximum absorption wavelength (ƛ_max_), particle size, and zeta potential (Additional file 1: Figure S3). The TEM image showed the spherical shape and the uniform distribution of these particles. The FTIR spectrum indicated the bands at 3425, 2919, 1601, 1400, 1253, and 1060 cm^−1^. The wave number at 3425 cm^−1^ was assigned to O–H stretch and weak band at 2919 cm^−1^ indicated the presences of alkane groups on the gold NPs surface [32]. The wavenumbers at 1601, 1400 cm^−1^ were attributed to C=C and C–C stretching vibrations, respectively. Moreover, the weak bands were formed at 1253 and 1060 cm^−1^ related to C–N stretched aromatic and aliphatic amines, respectively [33]. The zeta potential and particle size of gold gatekeepers were found around -17.66 mV and 7.92 ± 1.04 nm, respectively and displayed a single absorption peak in the visible range at 520 nm. After aggregation of gold capped NPs on the core–shell silica pores, a number of functional groups were detected. In this context, the absorption peaks at 1618 and 1210 cm ^−1^ were assigned to C=C and C–N, respectively. Furthermore, the zeta potential and particle size were determined around − 15.66 ± 0.82 mV and 39.71 ± 5.45 nm, respectively, and Au element was also indicated by EDX elemental analysis (Table 2). Pore-capping with gold NPs, N_2_ absorption/desorption isotherms still showed a type IV isotherm with type-H3 hysteresis loop, but their pore volume and surface area were markedly shifted to 0.4 cm^3^/g and 117.93 m^2^/g, respectively (Fig. 2A, B). The decreased surface area of the Au-NPs@DOX with BET results along with reduction in BJH pore size in comparison with SPION@MSNs confirmed that the gold gatekeepers have entirely capped the core–shell silica pores. Incorporation of heterofunctional PEG on the surface of Au-NPs@DOX was further proved by a weak band at the wave number of 1414 cm^−1^ in the FTIR spectrum indicating the presence of –COOH stretched carboxyl groups [34]. Moreover, the size increase (~ 20 nm), negative zeta potential, and reduced saturated magnetization value of PEG-Au-NPs@DOX may be attributed to the successful PEG coating. Upon EpCAM aptamer conjugation, a weak peak emerged at 2551 cm^−1^ assigned to the –SH group [35], thus verifying the effective decoration of EpCAM aptamer targeting on the nanocarriers surfaces. The mean zeta potential and particle size of final nanoconstructs were determined around − 19.79 ± 2.18 mV and 58.22 ± 8.54 nm, respectively. The successful conjugation of EpCAM aptamer on the surface of PEG-Au-NPs@DOX was further indicated by agarose gel electrophoresis. As seen in Fig. 2D, free EpCAM aptamer showed a band at the molecular weight of 50 bp, while PEG-Au-NPs@DOX did not show any band and Apt-PEG-Au-NPs@DOX stayed practically at the origin. Furthermore, TGA technique revealed a gradual increase in weight loss after each surface modification step (Fig. 2E). The weight loss values of SPION@MSNs, SPION@MSNs-NH_2_, Au-NPs@DOX, PEG-Au-NPs@DOX, and Apt-PEG-Au-NPs@DOX were 17.34%, 20.18%, 58.06%, 60.4%, and 62.79% respectively, indicating the successful modification of each step.

**Table 1:** Characterization parameters of the prepared nanocarriers

Samples	Zeta potential (mV)	Particle size (nm)	BET surface area (m^2^/g)	Pore volume (cm^3^/g)	BJH pore diameter (nm)
SPION@MSNs	− 19.52 ± 1.85	24.37 ± 0.21	432.79	1.23	1.21
SPION@MSNs-NH_2_	+ 15.35 ± 3.23	27.44 ± 1.83	ND	ND	ND
NPs@DOX	− 10.55 ± 1.00	32.38 ± 3.33	ND	ND	ND
Au-NPs@DOX	− 15.66 ± 0.82	39.71 ± 5.45	117.93	0.4	1.21
PEG-Au-NPs@DOX	− 18.50 ± 1.22	50.28 ± 4.76	ND	ND	ND
Apt-PEG-Au-NPs@DOX	− 19.79 ± 2.18	58.22 ± 8.54	ND	ND	ND

Particle size was measured by dynamic light scattering (DLS). Data are expressed as mean ± SD

*BET* Brunauer–emmett–Teller, *BJH* Barrett–Joyner–halenda, *SPION* Superparamagnetic iron oxide nanoparticle, *MSN* Mesoporous silica nanoparticle, *DOX* Doxorubicin, *PEG* Polyethylene glycol, *Apt* Aptamer, *ND* not determined

**Table 2:** Energy-dispersive X-ray (EDX) spectroscopy analysis of prepared nanocarriers

Samples	Si	Fe	N	C	O	Au
SPION@MSNs	35.21	2.58	0	6.63	55.58	0
SPION@MSNs-NH_2_	24.87	1.83	8.35	8.89	56.06	0
Au-NPs@DOX	7.96	0.69	5.72	16.84	27.75	41.05
PEG-Au-NPs@DOX	17.99	1.17	7.33	26.43	45.06	2.02

The data are represented as weight percentage (W%)

*SPION* Superparamagnetic iron oxide nanoparticle, *MSN* Mesoporous silica nanoparticle, *DOX* Doxorubicin, *PEG* Polyethylene glycol, *Apt* Aptamer

### Drug release profile

Intelligent release of cargoes at the region of interest is one of the most important steps to improve drug delivery behavior. For this aim, drug release experiments were designed in both acidic and physiological pH, which were adjusted to 5.4 and 7.4, respectively. As shown in Fig. 2F, the highest DOX release was detected at acidic pH which is similar to the endosomes and was much faster than those at physiological pH. Moreover, the amount of DOX released from Au-NPs@DOX in acidic buffer was about 40.43% which was significantly higher than that at physiologic condition. In this regard, the results indicated that only 3.9% of DOX was released from the Au-NPs@DOX in neutral medium over 96 h.

### Blood hemolysis evaluation

**Fig. 3:**
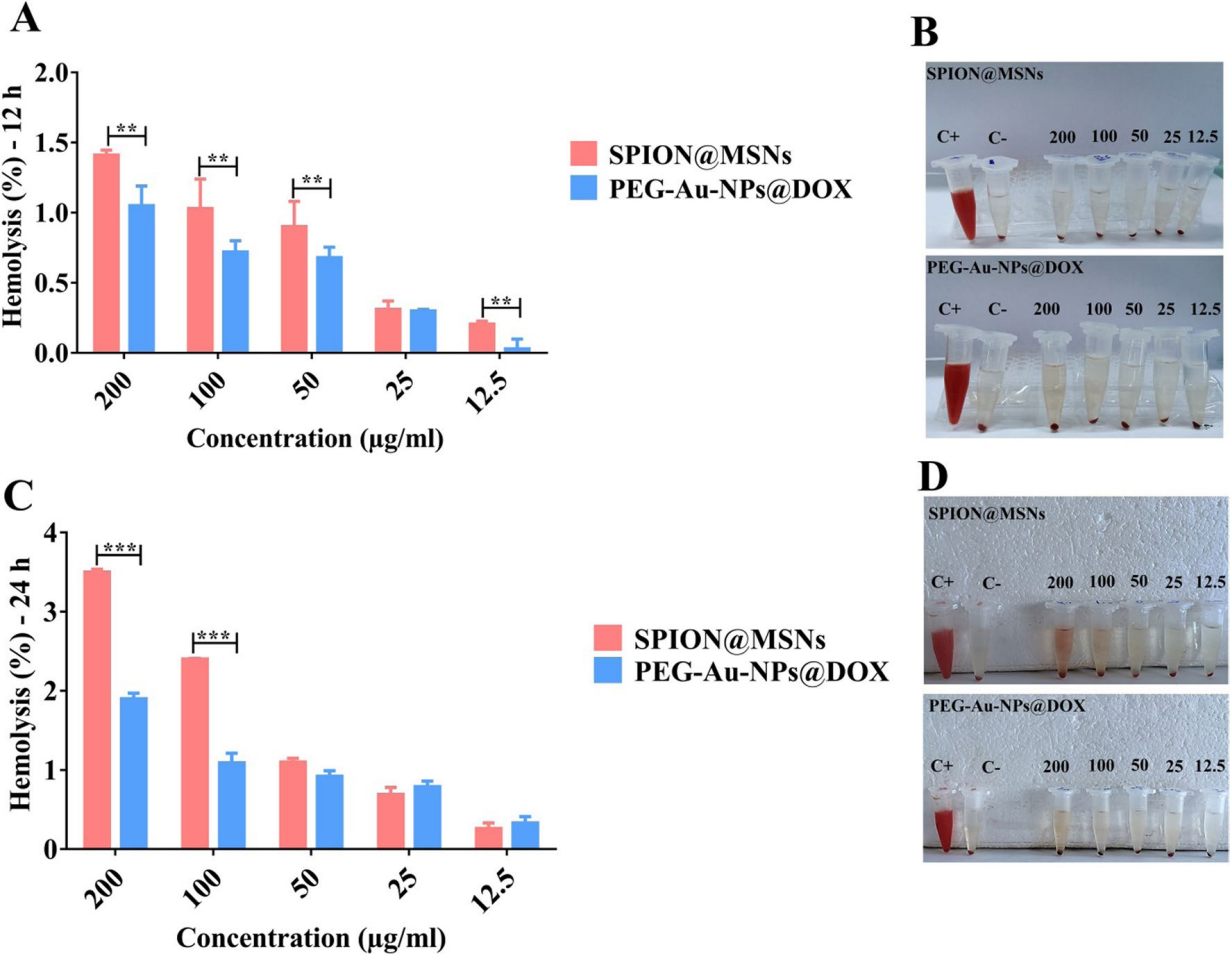
Hemolysis assay on SPION@MSNs and PEG-Au-NPs@DOX nanocarriers. Comparison of hemolytic activity in red blood cells (RBCs) following **A** 12 and **C** 24 h incubation with various concentrations (12.5–200 μg/ml) of SPION@MSNs and PEG-Au-NPs@DOX at 37 °C. Data are expressed as mean ± SD, n = 3, ** *p* < 0.01 and *** *p* < 0.001. Images of RBCs treated with mentioned concentration at **B** 12 h and **D** 24 h. Positive control (distilled water, C^+^), negative control (PBS, C^−^), and nanocarrier suspensions (200, 100, 50, 25 and 12.5, μg/ml) are indicated. *SPION* Superparamagnetic iron oxide nanoparticle, *MSN* Mesoporous silica nanoparticle, *PEG* Polyethylene glycol, *NP* nanoparticle, *Apt* Aptamer, *DOX* Doxorubicin

Covering the surface of nano-delivery systems with PEG as a well-known biodegradable polymer can greatly improve their behavior in the biological environments [18]. A hemolysis assay was conducted on PEG-Au-NPs@DOX and compared with the backbone in order to evaluate their biocompatibility in the blood. As shown in Fig. 3, all tested concentrations (12.5–200 μg/ml) of PEGylated nanocarriers resulted in less than 2% hemolysis at 12 and 24 h, demonstrating non-hemolytic activity. However, the hemolytic activity level of SPION@MSNs in RBCs reached to 3.5% and 2.4% at concentrations of 200 and 100 μg/ml, respectively, at 24 h. It seems that, PEG-modification reduces hemolytic activity and gives an excellent biosafety for intravenous injection.

### In vitro cytotoxicity

**Fig. 4:**
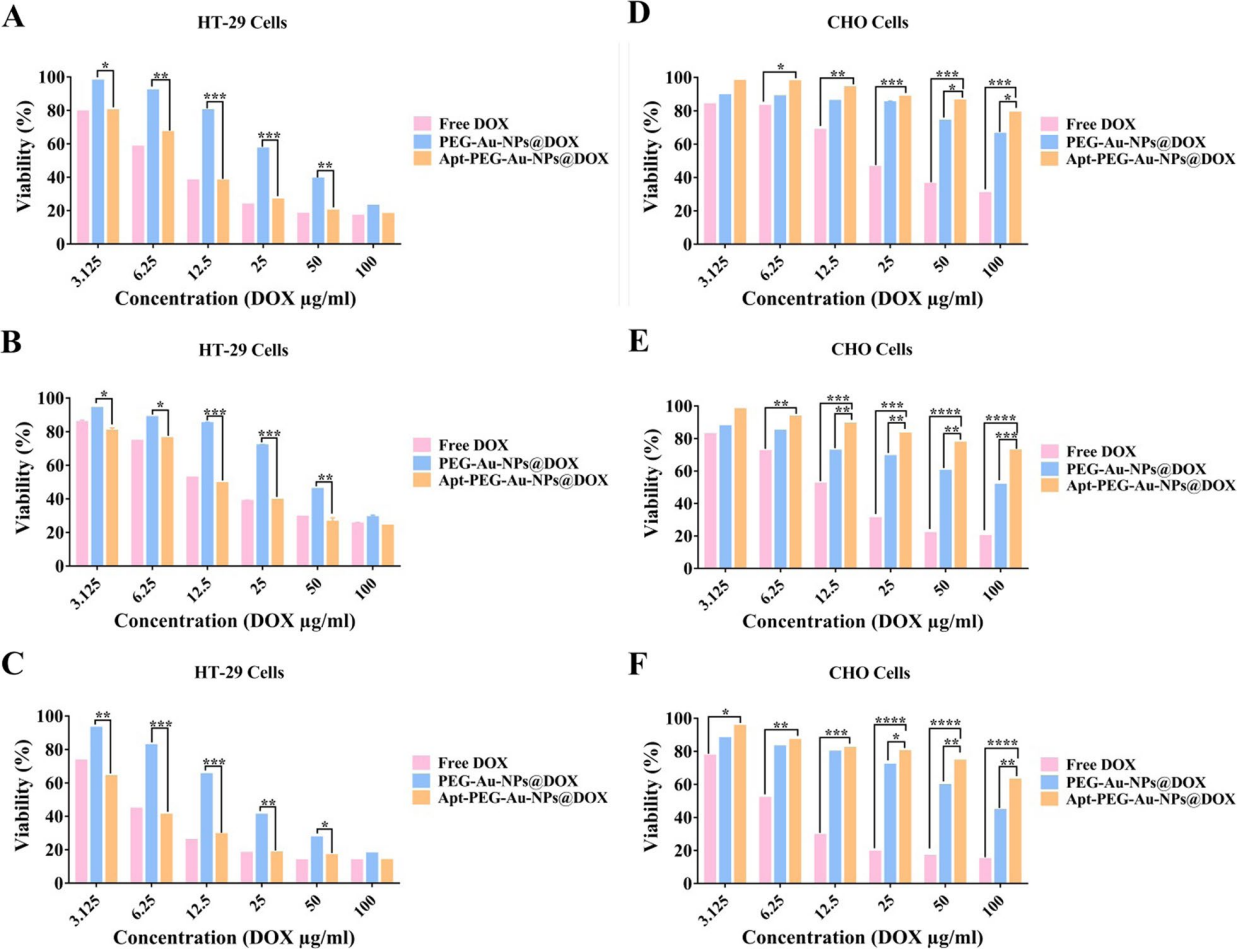
Evaluating the cytotoxicity and specificity of different formulations in vitro. Cytotoxic effects of free DOX, PEG-Au-NPs@DOX and Apt-PEG-Au-NPs@DOX were compared against EpCAM positive HT-29 cells for **A** 24, **B** 48 and **C** 72 h or against EpCAM negative CHO cells for **D** 24, **E** 48 and **F** 72 h. Data are expressed as mean ± SD. * *p* < 0.05, ** *p* < 0.01, *** *p* < 0.001 and **** *p* < 0.0001. *DOX* Doxorubicin, *PEG* Polyethylene glycol, *NP* nanoparticle, *Apt* Aptamer, *EpCAM* Epithelial cell adhesion molecule, *HT-29 cells* Human colorectal adenocarcinoma cells, *CHO cells* Chinese hamster ovary cells

In present study, we assessed the effects of conjugating EpCAM aptamer to nanocarriers, on their specificity to target EpCAM-expressing cells. The cytotoxicity of SPION@MSNs was first evaluated on both HT-29 and CHO cells to confirm the safety and nontoxicity of the backbone (data not shown). As shown in Fig. 4, there was a significant difference between toxicity of Apt-PEG-Au-NPs@DOX and PEG-Au-NPs@DOX as non-targeted formula on the EpCAM positive HT-29 cells in the range of 3.125–50 µg/ml, at 24, 48, and 72 h. Moreover, there was no significant difference between free DOX and targeted nanocarriers on HT-29 cells. For CHO cells (EpCAM negative cells), Apt-PEG-Au-NPs@DOX exhibited negligible toxicity in comparison with free DOX and PEG-Au-NPs@DOX. Furthermore, the IC_50_ values of free DOX and different formulations on both cell types following 24, 48, and 72 h treatments are calculated and presented in Table 3.

**Table 3:** IC_50_ values of free DOX and nanocarriers on HT-29 and CHO cells during 24, 48, and 72 h of treatments

Treatments	IC_50_ (µg/ml) ± SD (HT-29 cells)			IC_50_ (µg/ml) ± SD (CHO cells)		
	24 h	48 h	72 h	24 h	48 h	72 h
Free DOX	8.86 ± 1.78	6.83 ± 1.06	4.89 ± 1.06	14.00 ± 1.07	9.63 ± 1.08	5.66 ± 1.06
PEG-Au-NPs@DOX	33.90 ± 1.07	25.38 ± 1.07	15.99 ± 1.05	102.2 ± 1.12	77.01 ± 1.10	54.94 ± 1.26
Apt-PEG-Au-NPs@DOX	9.72 ± 1.08	7.29 ± 1.09	5.52 ± 1.09	320.5 ± 1.24	243.2 ± 1.27	179.2 ± 1.29

*SPION* Superparamagnetic iron oxide nanoparticle, *MSN* Mesoporous silica nanoparticle, *DOX* Doxorubicin, *PEG* Polyethylene glycol, *Apt* Aptamer

### Evaluating the effects of EpCAM aptamer on cellular internalization

**Fig. 5:**
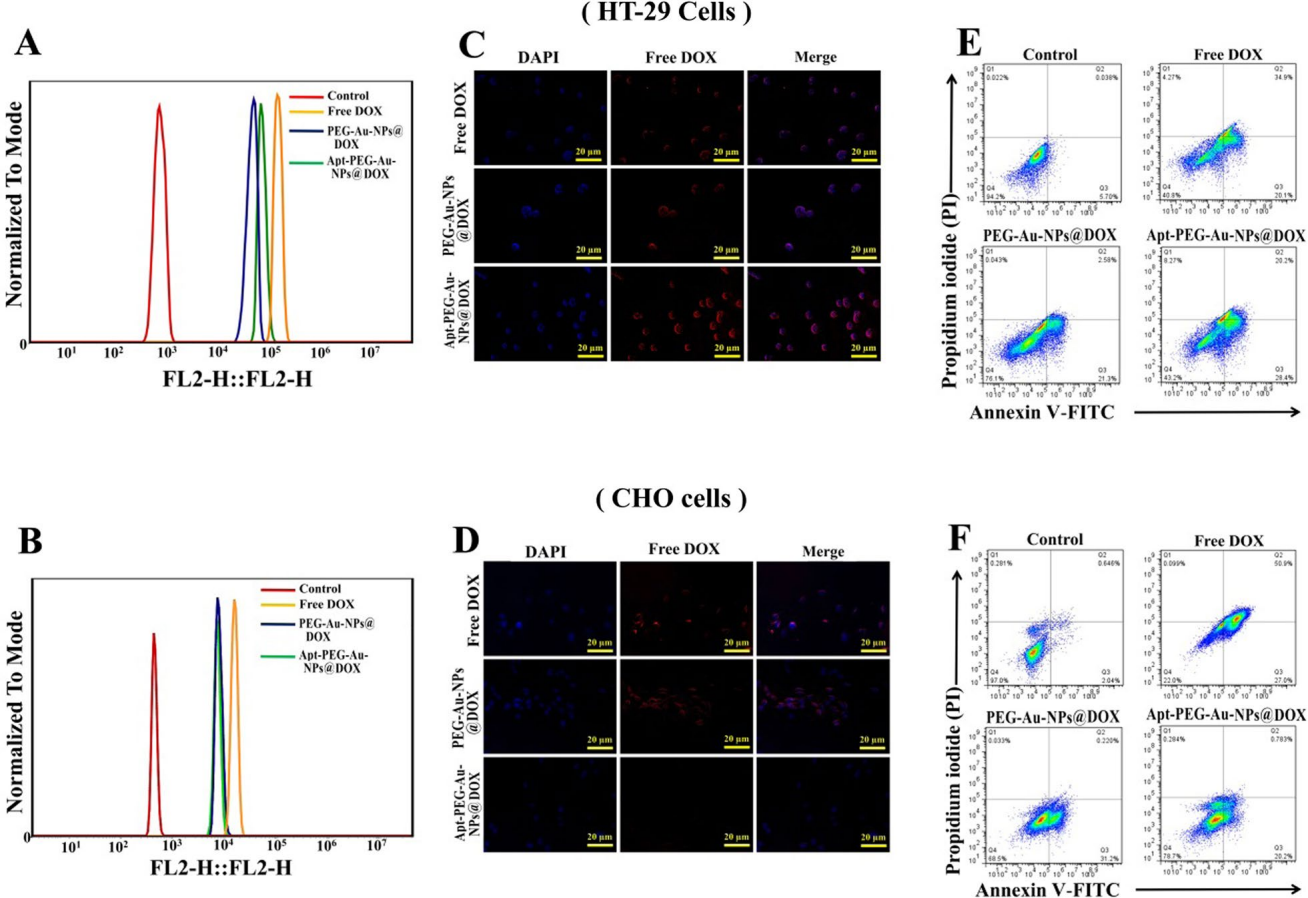
Cellular internalization and cell death mechanism analysis. The cellular uptake of free DOX and nanocarriers was investigated by both flow cytometry and fluorescence microscopy. HT-29 and CHO cells were treated with free DOX, PEG-Au-NPs@DOX and Apt-PEG-Au-NPs@DOX (DOX final concentration was 5 μg/ml) for 6 h at 37 °C. Flow cytometry histograms of **A** HT-29 and **B** CHO cells after treatment with free DOX, PEG-Au-NPs@DOX and Apt-PEG-Au-NPs@DOX. The results of cell internalization of free DOX and nanocarriers on **C** HT-29 and **D** CHO cells as visualized by fluorescent microscopy. Nuclei were stained with DAPI. Scale bar = 20 μm. Cell death mechanism was assessed by Annexin V-FITC/PI staining using flow cytometry. The results showed that free DOX, PEG-Au-NPs@DOX and Apt-PEG-Au-NPs@DOX induced apoptotic pathway in **E** HT-29 and **F** CHO cells. Viable, early, and late apoptotic cell populations accumulated in Q4, Q3, and Q2, respectively. *DOX* Doxorubicin, *SPION* Superparamagnetic iron oxide nanoparticle, *MSN* Mesoporous silica nanoparticle, *PEG* Polyethylene glycol, *NP* nanoparticle, *Apt* Aptamer, *EpCAM* Epithelial cell adhesion molecule, *HT-29 cells* Human colorectal adenocarcinoma cells, *CHO cells* Chinese hamster ovary cells

To assess the role of EpCAM aptamer on targeted cellular internalization of nanocarriers, we used both EpCAM positive and EpCAM negative cells. Tracking cellular uptake was evaluated using both flow cytometry and fluorescence microscopy. The cellular internalization by flow cytometry technique indicated higher uptake of Apt-PEG-Au-NPs@DOX in HT-29 cells as compared with PEG-Au-NPs@DOX, indicating the specific interaction between EpCAM aptamer and its receptor (Fig. 5A). Moreover, the fluorescent intensity of Apt-PEG-Au-NPs@DOX was lower in EpCAM^−^ CHO cells (Fig. 5B). It should be noted that, there was no significant difference for DOX uptake in HT-29 and CHO cells due to its unspecific passive entrance through lipid bilayer. These observations were further confirmed by fluorescence microscopy. As shown in Fig. 5C, D the internalization of the free DOX, PEG-Au-NPs@DOX and Apt-PEG-Au-NPs@DOX are clearly observable from the red fluorescence of DOX molecules. In addition, internalization of targeted nanocarriers in HT-29 cells showed stronger red fluorescence compared to CHO cells, confirming the EpCAM aptamer mediated endocytosis of Apt-PEG-Au-NPs@DOX nanocarriers.

### Assessment of cell death mechanism

To investigate the mechanism of cell death induced by free DOX, PEG-Au-NPs@DOX and Apt-PEG-Au-NPs@DOX in both HT-29 and CHO cells, Annexin V-FITC/PI staining was performed. Our data revealed that the viable cell population (Q4) markedly decreased to 40.8%, 76.1%, and 43.2% in free DOX, non-targeted and targeted nonocarriers-treated HT-29 cells compared with 94.2% in the control cells. Moreover, the percentage of early and late apoptotic cells (Q_2_ + Q_3_) increased from 5.7% in control to 55%, 23.8% and 48.6% in free DOX, PEG-Au-NPs@DOX and Apt-PEG-Au-NPs@DOX groups, respectively when treated with the equivalent amounts of 5 μg/ml DOX for 48 h (Fig. 5E). On the other hand, the percentage of Q2 + Q3 in CHO cells was 77.9% for free DOX, while for PEG-Au-NPs@DOX and Apt-PEG-Au-NPs@DOX it was about 31.4% and 20.9%, respectively (Fig. 5F). The notable viable CHO cell populations following treatment with Apt-PEG-Au-NPs@DOX in comparison with free DOX confirmed that the aptamer conjugation had a critical role in reducing the toxicity of DOX against healthy cells and EpCAM aptamer can efficiently eradicate EpCAM positive HT-29 cells via inducing apoptotic cell death mechanism.

### In vivo anti-tumor activity of nanocarriers

**Fig. 6:**
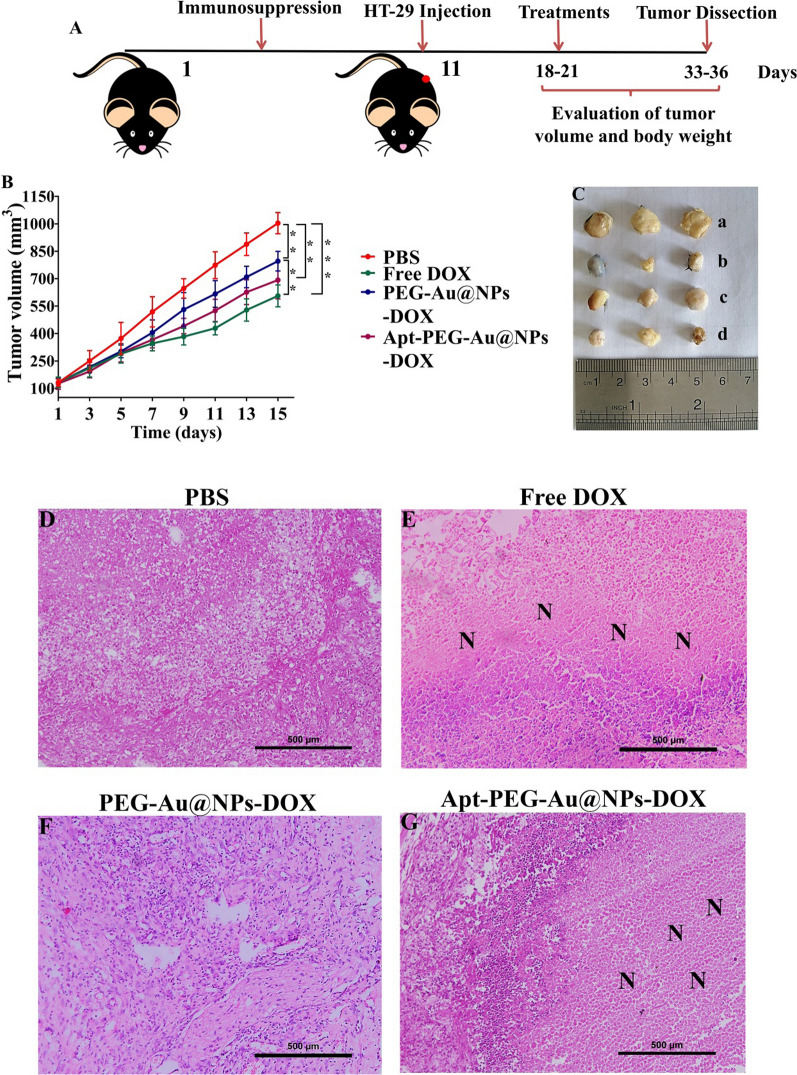
In vivo anti-tumor evaluation of free DOX, PEG-Au-NPs@DOX and Apt-PEG-Au-NPs@DOX (DOX final concentration was 1 μg/ml) in C57BL/6 mice bearing HT-29 tumors. **A** Schematic illustration of the therapy regimen. **B** Tumor growth curves showed strongest inhibition of free DOX and Apt-PEG-Au-NPs@DOX. Data are expressed as mean ± SD, n = 5. ** *p* < 0.01 and *** *p* < 0.001. **C** Tumor images in different experimental groups of (a) PBS, (b) free DOX, (c) PEG-Au-NPs@DOX and (d) Apt-PEG-Au-NPs@DOX treatments at day 15 post treatment. **D** H&E staining of tumor tissues indicated high levels of necrotic cells in both free DOX and targeted nanocarries. Scale bar = 50 μm. “N” represents necrotic areas within the tumor mass. *SPION* Superparamagnetic iron oxide nanoparticle, *MSN* Mesoporous silica nanoparticle, *PEG* Polyethylene glycol, *NP* nanoparticle, *Apt* Aptamer, *DOX* Doxorubicin, *H&E* Hematoxylin and eosin

After administration of immunosuppression protocol in C57BL/6 mice, tumors were induced by injecting HT-29 cells, and the feasibility of nanocarriers for CRC therapy was investigated (Fig. 6A). As shown in Fig. 6B, tumor volume in the control group was markedly elevated by time and all treatment groups could effectively reduce tumor growth. The results indicated that free DOX and Apt-PEG-Au-NPs@DOX caused the strongest inhibition in tumor growth. Furthermore, the non-targeted group, receiving PEG-Au-NPs@DOX, exhibited a significantly lower tumor growth inhibition as compared with free DOX group (*p* < 0.01). The tumor size of free DOX and Apt-PEG-Au-NPs@DOX treated groups was notably smaller than those receiving PBS at the end of treatment (Fig. 6C). H & E staining of the tumor tissues was carried out to further evaluate the anti-tumor activities of nanocarriers and showed the highest degree of tumor necrosis in both free DOX and Apt-PEG-Au-NPs@DOX treatment groups (Fig. 6D–G). These results confirm that the targeted nanocarriers had considerably higher anti-tumor properties in comparison with non-targeted formula owing to their tumor targeting potency.

### Biosafety evaluation

**Fig. 7:**
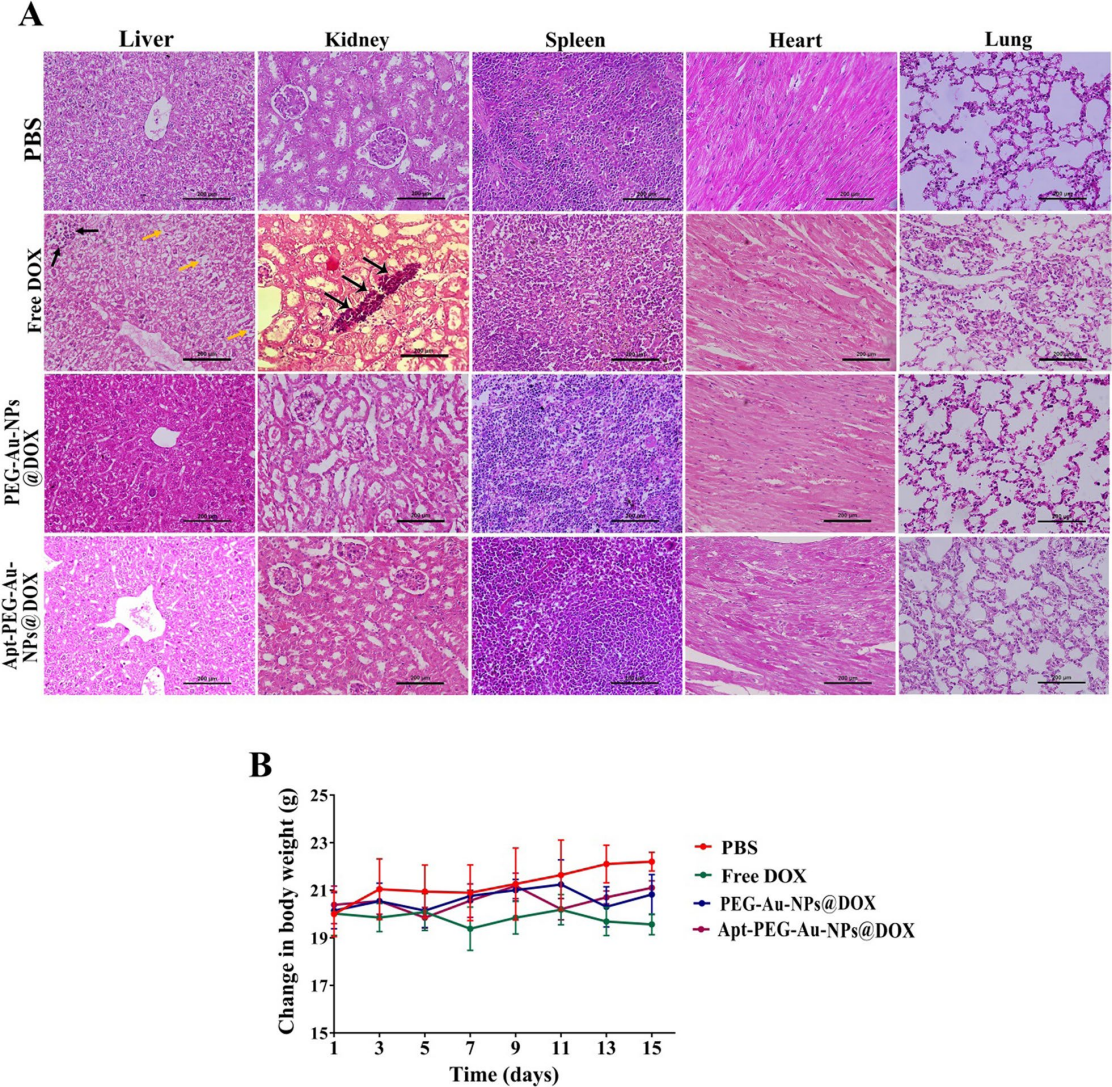
In vivo possible side effects evaluation. **A** H&E staining of main organs (liver, kidney, spleen, heart, and lung) collected on day 15 after treatments. Local accumulation of inflammatory cells in the liver and kidney (black arrows) were noticed in free DOX treated group. Moreover, vacuolar degeneration of hepatocytes associated with sinusoidal dilatation and congestion (yellow arrows) were detected in that group. Scale bar: 200 μm. **B** Body weight changes of immunocompromised C57BL/6 mice bearing human HT-29 cells during various treatments (n = 5). *SPION* Superparamagnetic iron oxide nanoparticle, *MSN* Mesoporous silica nanoparticle, *PEG* Polyethylene glycol, *NP* nanoparticle, *Apt* Aptamer, *DOX* Doxorubicin, *H&E* Hematoxylin and eosin

**Fig. 8:**
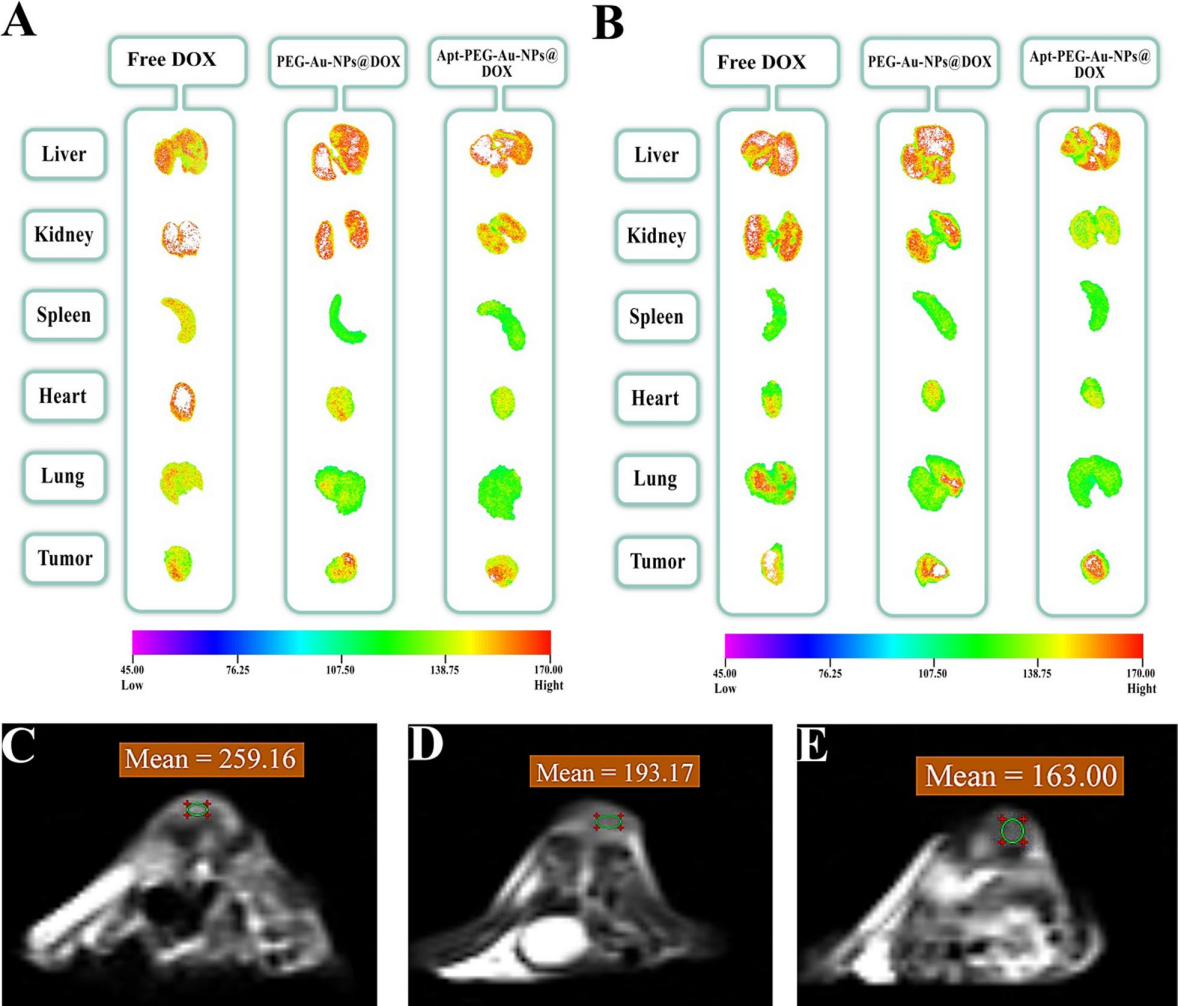
Representative ex vivo fluorescence and magnetic resonance imaging (MRI). Fluorescence images taken for main organs (liver, kidney, spleen, heart, and lung) and tumor at **A** 12 and **B** 24 h after injection of free DOX, PEG-Au-NPs@DOX and Apt-PEG-Au-NPs@DOX (DOX final concentration was 1 mg/kg). The T2-weighted coronal and axial images of the HT-29 tumor bearing C57BL/6 mice 12 h post injection of **C** PBS, **D** PEG-Au-NPs@DOX and **E** Apt-PEG-Au-NPs@DOX. The areas of the tumor are marked with a circle. *SPION* Superparamagnetic iron oxide nanoparticle, *MSN* Mesoporous silica nanoparticle, *PEG* Polyethylene glycol, *NP* nanoparticle, *Apt* Aptamer, *DOX* Doxorubicin

H&E staining of major organs including liver, kidney, spleen, heart, and lung, evaluating body weight and biodistribution of nanocarriers were performed to assess possible side effects. There were no obvious histological abnormalities in critical organs (Fig. 7A) and no noticeable changes in body weights (Fig. 7B) following treatment with non-targeted and targeted nanocarriers. In contrast, a remarkable decrease in body weight and local accumulation of inflammatory cells in the liver and kidney (black arrows) were noticed in free DOX treated group. It should be noted that, vacuolar degeneration of hepatocytes associated with sinusoidal dilatation and congestion (yellow arrows) were detected in the free DOX treated group. Furthermore, after intravenous injection of free DOX, PEG-Au-NPs@DOX and Apt-PEG-Au-NPs@DOX (DOX final concentration was 1 mg/kg), the mice were sacrificed at certain time points, 12 and 24 h post injection, and major organs were harvested to evaluate nanocarriers biodistribution by in vivo imaging system (IVIS). The obtained results showed that free DOX was mainly accumulated in the liver, kidneys, heart, and tumor after 12 h and its fluorescence signal was more intensified for lung tissue at 24 h. PEG-Au-NPs@DOX and Apt-PEG-Au-NPs@DOX were also captured in the liver and kidneys at 12 h. Interestingly, 24 h post injection, the distribution of targeted nanocarriers was less than non-targeted form in the kidney and lung, but more accumulated in the tumor tissue. Taken together, our results showed that Apt-PEG-Au-NPs@DOX was strongly distributed in the tumor with relatively low fluorescence intensity in normal tissues in comparison with free DOX indicating severe side effects (Fig. 8A, B).

### In vivo MRI

SPION as an MRI contrast agent was conjugated at the center of nanocarriers to evaluate the imaging potential of prepared nanocarriers. T2 weighted MRI scans on C57BL/6 mice bearing HT-29 tumors were performed at 12 and 24 h post-administration of PEG-Au-NPs@DOX and Apt-PEG-Au-NPs@DOX. Based on quantitative analysis, MRI signal intensity value of targeted nanocarriers was markedly lower than non-targeted formula both at 12 and 24 h post injection due to the high concentration of SPION within the tumor tissue (Additional file 1: Table S1). Moreover, the signal intensities measured on MRI, confirmed high distribution of the Apt-PEG-Au-NPs@DOX at the tumor region in comparison with PEG-Au-NPs@DOX at 12 h after the injection (Fig. 8C–E). It should be noted that there was no significant differences between 12 h, and 24 h of MRI scans; therefore, the images of 12 h are only reported. The encouraging MRI results confirmed that EpCAM aptamer mediated drug delivery to the malignant tissue through receptor-mediated mechanism.

## Discussion

Globally, CRC accounts for the second cause of cancer related mortality both in women and men [1]. Nanotechnology based DDSs provide a powerful treatment package which can better suppress cancer cell proliferation and metastasis. There are a series of inorganic NPs such as polyoxometalates (POMs), covalent organic frameworks (COFs), metal organic frameworks (MOFs), SPIONs, and MSNs with considerable potentials in the treatment of CRC as evidenced by in vitro and in vivo studies. Various reports on POM applications in CRC therapy have displayed encouraging anti-cancer activity [36–40]. However, their long-term cytotoxicity on normal cells [41], non-specific interactions with biomolecules [42], their high negative charge [43], and lower thermodynamic and kinetic stability [44] are remarkable drawbacks that so far have prevented the practical applications of POMs. COFs and MOFs are new classes of porous materials, which have also received a great attention as efficient nanocarriers for drug delivery applications. However, they still face some challenges including their toxicity, biodistribution, fate, and exertion which need to be well-defined prior to their further employment [20]. SPIONs have also been greatly attained owing to the unique magnetic properties for theranostic applications. These NPs may not be fully considered as nanocarriers due to low drug capacity and their toxicity concerns [45]. MSNs with distinctive physicochemical properties as mentioned earlier are attractive candidates for drug delivery applications. Particularly, silica which has been considered as ‘‘generally recognized as safe” (GRAS) by the United States Food and Drug Administration (FDA) [46]. We previously described various approaches to optimize physicochemical properties of DDSs as well as considering specific cancer cell receptors in the TME, which are two critical parameters that could significantly impact on drug delivery performance [20]. In this study, we used SPION as an MRI contrast agent within the MSN to provide a dual system for diagnosis and treatment in one-single nano-delivery platform. Generally, theranostic nanocarriers as current trend in cancer research have had a great impact in therapeutic field due to monitoring therapeutic efficacy during treatment and assessing possible side effects [47]. To this aim, we synthesized a novel theranostic platform for CRC therapy based on SPION@MSN which was further modified with gold gatekeepers, PEG, and EpCAM aptamer to increase therapeutic effects of DOX and decrease its severe side effects. We first synthesized spherical SPION@MSN nanocarriers with a size of ~ 20 nm in diameter and negative zeta potential. After introducing amine groups, DOX was successfully encapsulated in the open channels of MSNs with EE% and LC% of 98.65% ± 0.88 and 49.79% ± 1.03, respectively. This high loading capacity of DOX is due to the open mesoporous structure of the MSNs confirming the results obtained by TEM analysis. In the next step, a formulation of NPs comprising gold gatekeepers were synthesized, characterized, and then used to block the pore entrances of MSNs to control the release of DOX. Presence of specific functional groups, detectable signal of Au atoms, changes of N_2_ absorption/desorption type, significant reduction of surface area, decrease in BJH pore size and significant weight loss strongly supported the successful capping of the MSNs. It was hypothesized that the electrostatic interactions between amine groups on the surface of MSNs and citrate groups on the gold gatekeepers under physiological condition are the main capping mechanism [16]. In order to evaluate the capping efficiency, DOX release profile was compared in the pH 5.4 and pH 7.4. The results showed that the DOX release in acidic pH was significantly higher than that in the physiological pH. In fact, protonation of amine groups in the citrate buffer can trigger dissociation of gold gatekeepers and subsequently lead to intelligent DOX release. As shown in Fig. 2F, two stages of drug release were observed from Au-NPs@DOX formulation in the citrate buffer. The first stage was rapid and burst within the first 6 h which might have been due to the quick diffusion of DOX from nanocarriers. The second stage of DOX release was slow and sustained in the next 24 h. It can be concluded that pH-sensitive gatekeepers were responsible for intelligent DOX release from DDSs which has a considerable impact on improving drug delivery behaviors. Analogously, Li et al*.* conjugated acid-labile acetal groups with gold gatekeepers in order to facilitate controlled release of cargo molecules from MSNs at low pH [14]. Similarly, Babaei et al*.* electrostatically assembled citrate-functionalized gold gatekeepers on the surface of MSNs for intelligent 5-FU release. Their results indicated that the release of 5-FU was augmented under acidic pH [16]. Coating the DDSs with PEG polymer was a routine method for ensuring solubility and dispersibility of nanocarriers, enhancing their circulatory half-life and blood biocompatibility [17]. In this study, heterofunctional PEG was used to establish thiol–Au linkage between PEG and gold gatekeeper resulting in improving drug delivery performance. The size of NPs was significantly increased from 39.71 ± 5.45 to 50.28 ± 4.76 nm and the saturated magnetization value was markedly repressed in comparison with SPION@MSNs after PEGylation. This might be attributed to decrease in the magnetic core size and growth of the particle size followed by surface coating with PEG. Similarly, it was demonstrated that magnetic properties of SPIONs significantly decreased after introducing PEG as a coating agent due to reduction of saturation magnetization value [48]. Moreover, amount of oxygen and carbon elements was obviously elevated and the weight loss was also increased to 60.4% in the same temperature range. This finding in line with other mentioned results indicated the successful conjugation of PEG on the surface of nanocarriers. In this regard, numerous investigations were conducted to improve physicochemical properties of MSNs via PEG coating. For instance, long blood circulation with significantly lower entrapment in the liver, spleen, and lung was reported after PEGylation of MSNs [49]. Indeed, Desai et al*.* demonstrated that grafting both polyethylene imine (PEI) and PEG onto the surface of MSNs was helpful to overcome the challenges in oral administration, which led to increased blood half-life circulation and penetration into intestinal epithelial cells [50]. Other consequences of PEGylation strategy include improving colloidal stability and repression of hemolytic properties of MSNs [51]. With regard to these reports, hemolytic activity of PEG-Au-NPs@DOX was compared with SPION@MSNs in the concentration range of 12.5 to 200 μg/ml (Fig. 3). Similar to other reports, covering MSNs with PEG did not induce any observable hemolysis effect even at the highest particle concentrations which is less than the threshold value of 2% [52] at 12 and 24 h. In contrast, SPION@MSNs exhibited slightly dose-dependent hemolytic behavior just at 24 h. Obtained results are consistent with other studies showing hemolysis activity of silica NPs towards RBCs [53–55] and PEGylation could completely eliminate serious biosafety concerns of prepared nanocarriers in the given concentration range. In the next step, interaction between amine group of EpCAM aptamer and carboxylic acid group of heterofunctional PEG resulted in formation of targeted nanocarriers. Attachment of EpCAM aptamer to the surface of core–shell nanocarriers led to emerging a weak peak at 2551 cm^−1^ and caused a negative shift in zeta potential due to the presence of the thiol group of the aptamer. Moreover, agarose gel electrophoresis was performed to evaluate whether the EpCAM aptamer could bind to the surface of the nanocarriers. As shown in Fig. 2D, free aptamer moved along with the 50 bp DNA marker, whereas Apt-PEG-Au-NPs@DOX remained in the well due to their heavy weight. This result confirmed the effective decoration of nanocarriers with EpCAM aptamer and formation of targeted formula with the average size of 58.22 ± 8.54 nm.

In order to validate the feasibility of prepared nanocarriers for drug delivery purposes, the anti-cancer efficacy of non-targeted (PEG-Au-NPs@DOX) and targeted (Apt-PEG-Au-NPs@DOX) formulas was compared both in vitro and in vivo in the second part of the study. It has been demonstrated that HT-29 exhibited a high EpCAM expression [56] in comparison with CHO cells which were negative for this marker [57, 58]; so HT-29 and CHO cells were chosen for in vitro studies. The anti-cancer efficacy of free DOX, PEG-Au-NPs@DOX and Apt-PEG-Au-NPs@DOX was investigated by MTT assay. As evident in Fig. 4, treatment with free DOX and both nanocarriers induced a time- and dose-dependent decrease in viability of HT-29 cells. Moreover, there was a significant difference between anti-cancer activity of non-targeted and targeted nanocarriers against HT-29 cells during 24, 48, and 72 h. Remarkable anti-cancer activity of Apt-PEG-Au-NPs@DOX against HT-29 cells beside the lack of notable cytotoxicity on CHO cells as compared with PEG-Au-NPs@DOX, emphasized the critical role of targeting moiety for specific recognition of receptors and selective delivery of anti-cancer drugs. On the other hand, we found that the free DOX was ineffective against CRC owing to the similar cytotoxic effects on EpCAM positive and negative cells which confirm its severe side effects. Our results are consistent with other reports demonstrating that arming nanocarriers with targeting moieties is an effective strategy to induce selective cytotoxicity in target cancerous cells. For instance, Sakhtianchi et al. reported significant difference (*p* < 0.05) between toxicity profile of DOX-PEG-MMSNs and DOX-APT-PEG-MMSNs. Their results indicated that targeted nanocarriers were more toxic in compression with non-targeted nanocarriers in case of MCF-7 cells [59]. Moreover, Siminzar et al*.* revealed higher cytotoxicity of targeted nanocarriers (DOX-SPION@SiO_2_-MUC-1) against MCF-7 cells as compared to the non-targeted structure (DOX-SPION@SiO_2_) [60]. Tracking cellular internalization of nanocarriers by quantitative and qualitative analyses demonstrated the higher uptake of Apt-PEG-Au-NPs@DOX in HT-29 cells, compared with CHO cells, further confirming the MTT results. Moreover, free DOX was quickly distributed into HT-29 and CHO cells without any specificity so it had the highest cellular uptake and toxicity among the nanocarriers. These results confirm that the presence of EpCAM aptamer on the surface of DDSs could augment the internalization process in the EpCAM positive cells and minimize off-target effects. Furthermore, the results of cell death mechanism clearly showed that Apt-PEG-Au-NPs@DOX selectively induced more apoptotic cell death in HT-29 as compared to PEG-Au-NPs@DOX which is consistent with cytotoxicity and uptake results (Fig. 5). It can be concluded that the arming of MSNs with EpCAM aptamer can improve drug delivery performance leading to markedly greater cytotoxicity, cellular uptake and increased apoptotic level in the CRC cells overexpressing EpCAM biomarker. In the past decade, development of targeted MSNs has had a great impact on specific cancer cell recognition and increasing the therapeutic efficacy. For instance, it has been shown that recruiting the AS1411 and mucin-1 (MUC-1) as conventional targeting aptamers on the surface of MSNs can significantly increase selective delivery of DOX and strong toxicity against MCF-7 breast cancer cells [59, 60]. Hyaluronic acid (HA) is another targeting ligand, which has been widely conjugated to the MSNs for specific recognition of CD44 overexpressing colon cancer cells [61–63]. Moreover, there have been several reports that combined EpCAM aptamer as targeting element with MSNs to enhance the cytotoxic effects of anti-cancer drugs against CRC cells. Towards this end, in vitro results of Xie et al*.* demonstrated that modification of MSNs with EpCAM aptamer could augment cellular uptake and increase cytotoxicity of the DOX on SW620 cells as compared with non-targeted MSNs [25]. Moreover, it has been shown that combination of DNA EpCAM aptamer with MSNs can be considered as a favorable drug delivery platform for CRC therapy [26, 64]. Eventually, an immunocompromised C57BL/6 mouse model was used to compare the therapeutic efficacy of nanocarriers and evaluate their possible side effects. In vivo results indicated that free DOX and Apt-PEG-Au-NPs@DOX could remarkably suppress tumor growth as compared with non-targeted group demonstrating high levels of tumor necrosis (Fig. 6). Biosafety evaluation indicated the body weight loss, tissue damages, and non-specific accumulation in normal tissues following the free Dox treatment; confirming its severe side effects. In this context, presence of EpCAM aptamer, as targeting ligand, in the treatment group could significantly decrease DOX toxicity as shown with no weight loss, lack of normal tissue damages, and significant intratumoral saturation (Figs. 7 and 8). Generally, EpCAM biomarker is closely associated with cancer cell proliferation [65] and migration [66], thus development of DDSs which target the EpCAM receptors may lead to reduction of cancer cell metastasis. In vivo data showed that Apt-PEG-Au-NPs@DOX presented better anti-cancer effects in comparison with PEG-Au-NPs@DOX. This could be attributable to passive accumulation of non-targeted nanocarriers at the tumor tissue while, targeted nanocarriers accumulated via both passive and active targeting. Generally, several studies have used different types of theranostic nanocarriers for therapeutic purposes. In order to get a comprehensive evaluation, the results were compared with other studies and summarized in Table 4. It can be concluded that prepared theranostic NPs with ~ 58 nm diameter must have circulated safely in the blood due to the presence of the PEG polymer on their surface and reached the TME through EPR effect. It has been shown that nanocarriers smaller than 100 nm can easily pass the vessel gaps in the TME [67] and we expect the designed nanocarriers to have effectively penetrated within the tumor. At this stage, incorporation of SPION as an MRI contrast agent makes it possible to track intratumoral accumulation of nanocarriers. EpCAM aptamer specifically interacts with EpCAM receptor in the TME and facilitates internalization of the targeted nanocarriers. Loaded DOX would be released into the cytosol under acidic environment and results in cancer cell death, while reducing the side effects.

**Table 4:** Examples of theranostic nanocarriers used for therapeutic purposes

Theranostic nanocarriers	Contrast agents	Particle size	Therapeutic agents	Targeting moieties	Cancer cell lines	Animal models	References
YVO4:Eu3 + @MSN	YVO4:Eu3 +	~ 375 nm	DOX	None	HeLa cells	None	[68]
YVO4:Eu3C and Fe _3_O_4_-MSN	YVO4:Eu3C	~ 50 nm	DOX	None	HeLa and MCF-7 cells	None	[69]
M-MSN	MnFe_2_O_4_	~ 100–150 nm	DOX	Folic acid	HeLa cells	Albino mice	[70]
pMMSN	Not used for imaging purpose	175.7 ± 11.4 nm	DOX	None	None	S180 tumor bearing mice	[71]
MMS	Not used for imaging purpose	~ 150 nm	DOX	None	HeLa cells	None	[72]
IONP	IONP	~ 4–10 nm	DOX	Folic acid	HeLa cells	None	[72]
MSN-IONP	IONP	60 ± 2 nm	None	None	LNCaP cells	LNCaP tumor bearing nude mice	[73]
Au@SPIONs	SPION	~ 19 nm	None	MUC-1 aptamer	L929 and HT-29 cells	None	[74]
GoMe	Gold nanoparticle	50.87 ± 10.69 nm	DOX	None	A2058 cells	Lung tumor bearing FVB mice	[75]
SPION	SPION	Not reported	None	Hyaluronic acid	MDA-MB-231 cells	MDA-MB-231 tumor-bearing mice	[76]
Au@MSN	Gold nanoparticle	~ 63.12 nm	5-FU	EpCAM aptamer	HepG2 cells	HepG2 tumor bearing nude mice	[16]
SPION	SPION	~ 58 nm	siRNA	Folic acid	SGC-7901 cells	None	[77]
MMSN	Not used for imaging purpose	18.68 ± 2.31 nm	Epi	None	C26 cells	C26 tumor bearing mice	[78]
SPION	SPION	Below 170 nm	DOX	Folic acid	HCT116 and MCF-7 cells	HCT116 tumor bearing mice	[79]
MSN-EuGd	EuGd	~ 120 nm	None	Hyaluronic acid and TAT peptide	A549 cells	None	[80]
MMSNs	SPION	89.88 ± 4.7 nm	DOX	AS1411 aptamer	MCF-7 cells	None	[59]
SPION@MSN	Not used for imaging purpose	~ 27–50 nm	DOX	MUC-1 aptamer	MCF-7 cells	None	[60]
HMSNsCS-CuS	CuS	150 ± 13 nm	DOX	None	MDA-MB-231 cells	MDA-MB-231 tumor bearing nude mice	[81]

*MSN* Mesoporous silica nanoparticle, *DOX* Doxorubicin, *M-MSN* Mesoporous silica-coated superparamagnetic manganese ferrite (MnFe_2_O_4_) nanoparticle, *pMMSN* Phosphonate-terminated magnetic mesoporous nanoparticle, *MMS* Magnetic mesoporous silica nanoparticle, *IONP* Iron oxide nanoparticle, *MUC-1* Mucin 1, *GoMe* Gold/Mesoporous silica hybrid nanoparticle, *EpCAM* Epithelial cell adhesion molecule, *MMSN* Magnetic mesoporous silica nanoparticle, *Epi* Epirubicin, *HMSNsCS-CuS* Mesoporous silica nanoparticles gated by chitosan-copper sulfide composites

## Conclusion

Several strategies need to be considered in designing nanocarriers to improve drug delivery performance and therapeutic outcomes. Overall, we developed a biocompatible, pH-sensitive, and targeted theranostic platform based on MSNs, which can effectively deliver DOX to human CRC cells, maximize anti-cancer activity, and minimize off-target toxicities. In this context, Apt-PEG-Au-NPs@DOX offered numerous advantages as a therapeutic platform in CRC therapy including (1) in vivo tracking, (2) high drug loading capacity, (3) biocompatibility, (4) intelligent and sustained drug release at acidic pH, (5) long blood circulation to passive accumulation at tumor site (6) appropriate size for tumor penetration (~ 58 nm), and (7) capability of targeting HT-29 tumors by active targeting mechanisms. The current multifunctional DDS constitutes a favorable replacement for CRC therapy, however further studies are required before it can reach the clinic.

## Experimental section

### Materials

N-hydroxysuccinimide (NHS), 1-ethyl-3-(3-dimethylaminopropyl) carbodiimide hydrochloride (EDC), n-cetyltrimethylammonium bromide (CTAB), Fe_3_O_4_, chloroauric acid (HAuCl_4_), trisodium citrate, tetraethyl orthosilicate (TEOS), (3-aminopropyl)triethoxysilane (APTES), and 4′,6-diamidino-2-phenylindole (DAPI) were purchased from Sigma-Aldrich Co. (Germany). Heterofunctional PEG polymer with a terminal thiol and carboxylic acid functional groups (SH–PEG–COOH, Mw: 3500) was purchased from GemChem (USA). Roswell Park Memorial Institute 1640 (RPMI 1640) medium, fetal bovine serum (FBS) and penicillin/streptomycin were purchased from Gibco (Scotland). FITC Annexin V apoptosis detection kit with propidium iodide (PI) was obtained from BioLegend (USA). Trypsin and 3-(4,5-dimethylthiazol-2-yl)-2, 5-diphenyltetrazolium bromide (MTT) were purchased from Tinab Shimi (Iran). Doxorubicin hydrochloride (DOX) was purchased from Euroasia Co. Ltd. (India). Matrigel® matrix (DLW354263) was obtained from Corning Inc. (USA). The 48 mer EpCAM DNA aptamer (sequence: 5′‐amine CACTACAGAGGTTGCGTCTGTCCCACGTTGTCATGGGGGGTTGGCCTG-3′-thiol) was synthesized by MicroSynth (Switzerland). DNA marker (50 bp), tris–borate-EDTA (TBE) buffer, and agarose powder were purchased from DENAzist Asia (Iran). Ethidium bromide was purchased from SinaClon (Iran). Furthermore, absolute ethanol, chemical reagents, and other solvents were obtained from Merck (Germany).

Human colon cancer cell line (HT-29) and Chinese hamster ovary (CHO) cell line were obtained from Pasteur Institute, Tehran, Iran and cultured in RPMI 1640 medium supplemented with 10% (v/v) FBS at 37 °C containing 5% CO_2_ in a humidified incubator.

### Synthesis procedures

#### Synthesis of magnetic mesoporous silica NPs (SPION@MSNs)

SPION@MSN core–shell nanocarriers were prepared according to the published method by Yang et al. [82]. Fe_3_O_4_ (200 mg) was dispersed in the mixture solution of ethanol (80 ml) and deionized water (20 ml) followed by drop wise addition of TEOS (1 ml) under nitrogen gas condition at 40 °C for 2 h. In the next step, magnetic NPs were separated using centrifugation (6000*g* for 15 min) and re-dispersed in a mixed solution containing deionized water (20 ml), NH_3_ (1 ml) and CTAB (0.75 g). The solution was heated up to 60 °C under vigorous stirring (100 rpm) followed by TEOS (2.5 ml) addition and preserved for 2 h under reflux condition. The resultant product was collected by centrifugation and washed three times with ethanol. In order to completely remove CTAB from MSNs, the calcination was performed at 600 °C for 5 h.

#### Synthesis of NH_2_-modified SPION@MSNs

SPION@MSN core–shell nanocarriers (16 mg) were dispersed in ethanol (16 ml), followed by the addition of APTES (60 µl) and the mixture was stirred at room temperature for 24 h. The SPION@MSNs-NH_2_ were collected by centrifugation (10,000*g* for 20 min) to remove excess APTES and solvent, followed by washing the mixture twice with ethanol [83].

#### Loading of DOX into the NH_2_-modified SPION@MSNs

SPION@MSNs-NH_2_ (2 mg) were suspended in 1 ml DOX solution and the mixture sonicated, and then stirred for 48 h at room temperature. Then, the resulting formula SPION@MSNs-NH_2_@DOX (that is abbreviated as NPs@DOX) was centrifuged (17,000*g* for 15 min) and the absorbance of supernatant (unloaded free DOX) was determined by ultraviolet–visible spectrophotometry (UV/Vis; Eppendorf, Germany) at 480 nm according to the standard curve of known concentrations of DOX solutions. Finally, DOX encapsulation efficiency (EE%) and drug loading capacity (LC%) were calculated as follows [84]:

$${\text{EE}}\% = \frac{{{\text{Total }}\upmu {\text{g of DOX}} -\upmu {\text{g of DOX in supernatant}}}}{{{\text{Total }}\upmu {\text{g of DOX}}}} \times 100$$

$${\text{LC}}\% = \frac{{{\text{Total }}\upmu {\text{g of DOX}} -\upmu {\text{g of DOX in supernatant}}}}{{{\text{Total }}\upmu {\text{g of nanocarriers}}}}{ } \times 100$$

#### Synthesis of gold capped NPs (Au-NPs@DOX)

Gold NP can be used as an intelligent gatekeeper to control the release of DOX from SPION@MSN. In this regard, freshly prepared HAuCl_4_ (300 ml; 0.5 mM) was heated with a heating mantle under vigorous stirring. When the temperature reached 80 °C, freshly prepared trisodium citrate solution (30 ml; 38.8 mM) was added into the aqueous solution of HAuCl_4_. The color of solution changed immediately to black gray and then to pink after 2–4 min. The solution was kept at 70 °C for 5 min and then cooled down while being gently stirred [85]. Gold NPs were investigated by transmission electron microscopy (TEM; Philips, Germany), Fourier-transform infrared spectrum (FTIR; Thermo, USA), UV/Vis spectroscopy, zeta potential measurements (CAD Instruments; France), and dynamic light scattering (DLS; Cordouan Technologies, France).

Prepared gold NPs (1 ml; 0.2 mM) were added to suspension of NPs@DOX (1 ml; 2 mg/ml) and stirred for 24 h at room temperature. The aggregation of gold capped gatekeepers on core–shell silica pores was found to depend strongly on electrostatic interactions between citrate and amine groups.

#### Synthesis of PEG-Au-NPs@DOX and Apt-PEG-Au-NPs@DOX

**Fig. 9:**
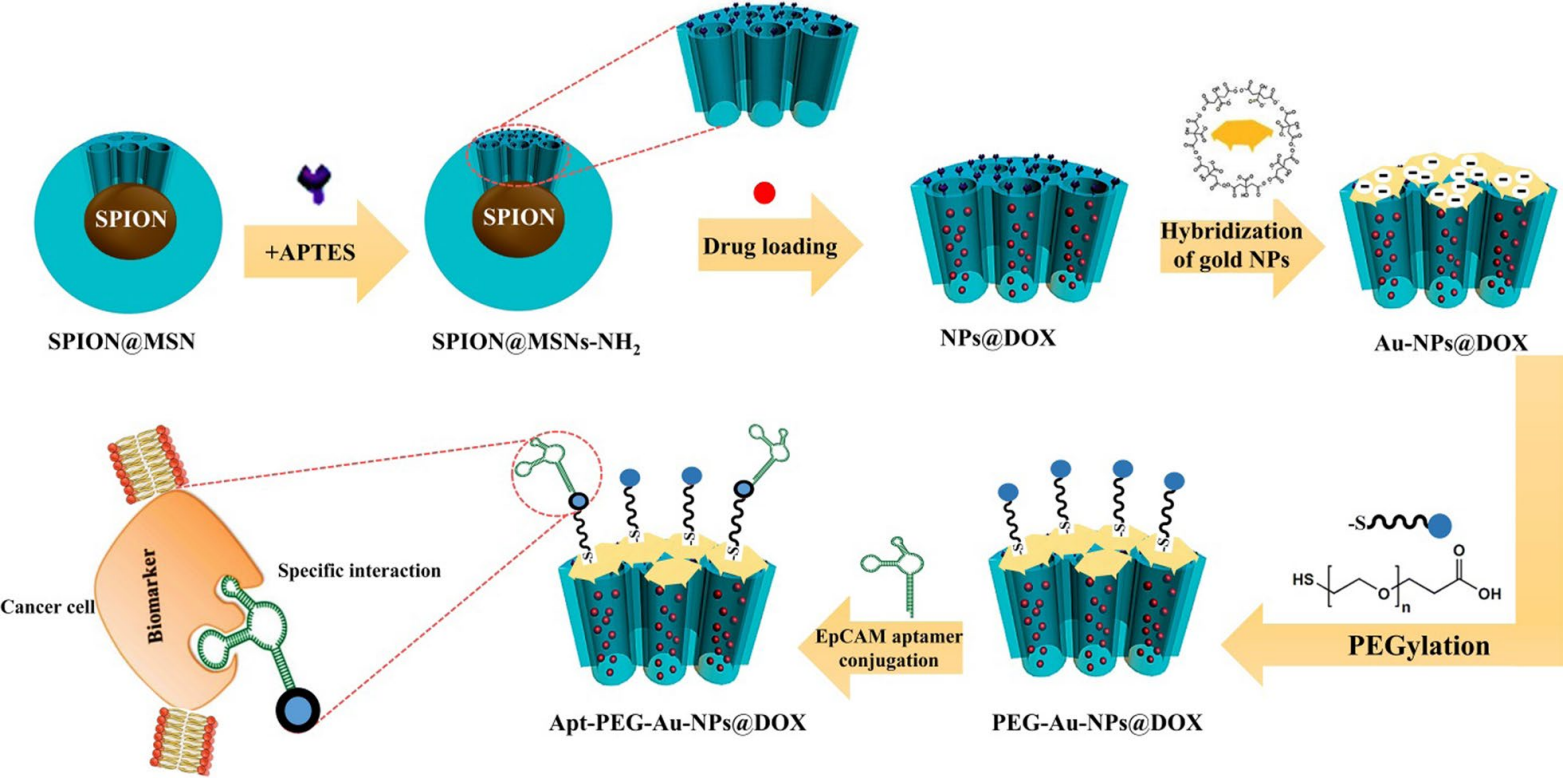
Schematic illustration of nanocarriers preparation. The surface charge of SPION@MSN core–shell nanocarrier was modified followed by the addition of APTES. In the next step, DOX was encapsulated in the open channels of MSNs and gold gatekeepers were used to control the release of DOX. Heterofunctional PEG (SH–PEG–COOH) and EpCAM aptamer were applied to prepare non-targeted and targeted nanocarriers, respectively. *SPION* Superparamagnetic iron oxide nanoparticle, *MSN* Mesoporous silica nanoparticle, *APTES* (3-Aminopropyl)triethoxysilane, *NP* Nanoparticle, *PEG* Polyethylene glycol, *EpCAM* Epithelial cell adhesion molecule, *Apt* Aptamer

6 mg of heterofunctional PEG (SH–PEG–COOH) was added into the suspension and allowed to react for 24 h at room temperature under vigorous stirring. The thiol group strongly binds to the surfaces of Au-NPs through Au–S linkage in order to prepare non-targeted nanocarriers. In the next step, amine functionalized EpCAM DNA aptamer was covalently attached to carboxylic group of PEG on the surface of PEG-Au-NPs@DOX utilizing EDS and NHS as activating agents. For this aim, EDS (3.27 mg) and NHS (1.96 mg) were added to the suspension to result in carboxylic acid groups activation, and then EpCAM aptamer (20 μl, 5 μM) was added to the suspension and stirred overnight at room temperature (Fig. 9). Finally, targeted Apt-PEG-Au-NPs@DOX were pelleted by centrifugation at 17,000*g* for 15 min and washed three times with deionized water.

### Physical characterization

The FTIR analysis of all samples was performed to confirm proper conjugation of the functional groups in each step. Morphology and size of the prepared NPs were evaluated by atomic force microscopy (AFM; BRUKER, USA), high-resolution transmission electron microscopy (HR-TEM; FEI, USA) with an accelerating voltage of 200 kV and field emission scanning electron microscopy (FESEM; TESCAN MIRA, Czech Republic) equipped with an energy‐dispersive X‐ray spectrometer operated at 30 kV. Elemental compositions of the nanocarriers (Si, Fe, N, C, O, and Au) were evaluated by energy-dispersive X-ray analysis (EDX; TESCAN MIRA, Czech Republic). The particle sizes and surface zeta potential values were measured by Zeta Compact Potential Analyzer. Specific surface areas, pore size distribution and pore volume of SPION@MSNs and Au-NPs@DOX were determined using the Brunauer–Emmett–Teller (BET) and Barrett–Joyner–Halenda (BJH) methods (BEL, Japan). The magnetic behavior of the SPION@MSNs and PEG-Au-NPs@DOX was assessed by using vibrating sample magnetometer (VSM; Lake Shore Cryotronics, Netherlands). The conjugation of EpCAM aptamer on the surface of PEG-Au-NPs@DOX was further checked by electrophoresis on agarose gel (2%) along with the DNA marker. The electrophoresis was carried out at 80 V for 40 min in TBE buffer. The gel was then stained with ethidium bromide (0.5 mg/ml) and imaged using gel documentation system (Major science, USA). Finally, thermogravimetric analysis (TGA; TA, USA) was performed at a heating rate of 20 °C/min in air to evaluate the thermal profile of all NPs.

### In vitro drug release

The pH-responsive drug release from Au-NPs@DOX was evaluated by dialysis membrane method. The mentioned NPs were dispersed in 3 ml phosphate-buffered saline (PBS; pH 7.4) and introduced into dialysis bags (cutoff = 1000 Da). Then, the sealed membrane was immersed in 30 ml of release medium (PBS; pH = 7.4 and citrate buffer; pH = 5.4) and incubated at 37 °C with shaking at 60 rpm for 96 h. In specific time points, release media (3 ml) were collected and replaced with the same volume of fresh release media to keep a constant volume. Eventually, the concentration of released DOX was determined by UV/Vis spectroscopy at 480 nm. All assessments were performed in triplicate.

### Hemolysis assay

Hemolysis test was developed to determine the potential cell lysis capacity of the prepared nanocarriers. For this purpose, human blood sample was obtained from a healthy donor and centrifuged (1500*g* for 10 min at 4 °C) to collect red blood cells (RBCs). After diluting pellets with PBS (1:10), different concentrations of SPION@MSNs and PEG-Au-NPs@DOX (12.5 to 200 μg/ml) were added to diluted samples and incubated at 37 °C in agitation at 100 rpm for 12 and 24 h. The mixtures were then centrifuged (2500*g* for 1 min), and the hemoglobin released in the supernatant was analyzed by enzyme-linked immunosorbent assay (ELISA; Awareness Technology, USA). To confirm the hemolysis assay, distilled water and PBS were used as positive and negative controls, respectively and hemolysis percentage was measured via the following equation:

$$\small {\text{Hemolysis}}\% = \frac{{{\text{absorbance of NPs}} - {\text{absorbance of negative control}}}}{{{\text{absorbance of positive control}} - {\text{absorbance of negative control}}}} \times 100$$

### In vitro cytotoxicity study

The cytotoxicity of prepared nanocarriers was assessed on HT-29 and CHO cells as EpCAM positive and negative cell lines, respectively, using MTT assay according to the Mosmann method with some modifications [86]. Cells were cultured at seeding density of 8 × 10^3^ cells/well in 96-well plates. The next day, the cell culture media were replaced and cell lines were treated with different concentration of free DOX, PEG-Au-NPs@DOX and Apt-PEG-Au-NPs@DOX (100 µg/ml to 1.56 µg/ml; equivalent concentration of DOX) in three replicates. After 24, 48 and 72 h of treatments, 20 µl of MTT solution (5 mg/ml in PBS) was added to each well and incubated for 4 h at 37 °C. Afterwards, the media were removed and 160 µl dimethyl sulfoxide (DMSO) was added to each well to dissolve the purple crystals. Finally, the optical density (OD) was measured at 540 nm using an ELISA reader and cell viability was compared with untreated cells, which were considered as 100% cell viability.

### In vitro cellular uptake

The cellular uptake of nanocarriers was investigated by both flow cytometry technique and fluorescence microscopy. For this purpose, HT-29 and CHO cells were seeded in 6-well plates at a density of 2 × 10^5^ cells/well and incubated for 24 h. Then, free DOX, PEG-Au-NPs@DOX and Apt-PEG-Au-NPs@DOX (final concentration of DOX was 5 μg/ml) were added to different wells and incubated for 6 h. Subsequently, the cells were trypsinized, centrifuged (400*g* for 15 min) and resuspended in 300 µl cold PBS (1X). The fluorescence intensity of cells was determined by a flow cytometer (BD Accuri C6, USA) in FL2 channel and related data were analyzed using FlowJo 7.6 software. Moreover, cellular uptake of nanocarriers was also observed by fluorescence microscopy. For this aim, after treatment of both cell types with mentioned concentrations of nanocarriers for 6 h, they were washed three times with PBS, fixed in 4% paraformaldehyde (15 min at 4 °C), and stained with DAPI for 10 min in the dark. After washing with PBS for three times, the cells were visualized under a fluorescent microscope (Olympus BX51, Japan).

### Studying cell death mechanism

To evaluate the mechanism of cell death induced by prepared nanoparticles, HT-29 and CHO cells were seeded with a density of 2 × 10^5^ cells/well in 6-well plates for 24 h. Cells were then treated with free DOX, PEG-Au-NPs@DOX and Apt-PEG-Au-NPs@DOX containing the equivalent amount of 5 μg/ml DOX for 48 h. Afterwards, cells were collected and stained with Annexin V-FITC kit with PI according to the manufacturer’s protocol. Finally, cells were subjected to flow cytometry to evaluate the mechanism of cell death induced by nanocarriers and the data were analyzed by FlowJo 7.6 software.

### In vivo studies

#### Evaluation of anti-tumor efficacy

The animal experiments were carried out following the guidelines approved by Animal Ethics Committee of Ferdowsi University of Mashhad (IR.UM.REC.1400.032). Immunosuppression of female C57BL/6 mice (4–6 weeks old) was performed as described previously [87]. 8 × 10^6^ HT-29 cells (suspended in 1:1; FBS: Matrigel) were subcutaneously injected at the back of the immunocompromised mice. When tumors reached approximately 100–150 mm^3^, mice were randomized into four different experimental groups (n = 5 per group) and intravenously treated with (1) PBS as control, (2) free DOX (1 mg/kg), (3) PEG-Au-NPs@DOX (1 mg/kg of DOX in 100 μl NPs) and (4) Apt-PEG-Au-NPs@DOX (1 mg/kg of DOX in 100 μl NPs) via the tail vein at days 1, 3, 6 and 9. The growth of tumors was measured using a digital caliper (Mitutoyo, Japan) every other day.

#### Evaluation of biosafety and biodistribution

In order to assess the possible side effects, the body weights were monitored every other day for up to 15 days. The mice were then sacrificed and the major organs including liver, kidney, spleen, heart, lung, and tumor were collected for histological analysis with hematoxylin and eosin (H&E) staining. Moreover, to investigate the distribution of nanocarriers, 100 μl of free DOX, PEG-Au-NPs@DOX and Apt-PEG-Au-NPs@DOX were injected at a concentration of 1 mg/kg DOX via the tail vein of immunocompromised mice bearing HT-29 tumors. The mice were then sacrificed at 12 and 24 h after injection, and the main mentioned organs were isolated for analysis by an in vivo imaging system (IVIS; KODAK, USA).

#### Magnetic resonance imaging (MRI)

MRI was conducted to evaluate the intratumoral accumulation of non-targeted and targeted nanocarriers [88]. When the tumor reached ~ 200–300 mm^3^, the mice were tail-vein injected with PBS (as a control group), PEG-Au-NPs@DOX and Apt-PEG-Au-NPs@DOX. The T2-weighted coronal and axial images of the tumor were performed under a 1.5 T MRI scanner (MAGNETOM symphony; SIEMENS, Germany) with following parameters: protocol = turbo spin echo (TSE); repetition time (TR) = 5000 ms; echo time (TE) = 91 ms; resolution = 384 × 384 pixel and slice thickness = 3 mm. Signal intensity measurements were conducted by DICOM viewer software (Medixant. RadiAnt DICOM Viewer [Software], Version 2020.2. Jul 19, 2020. URL: https://www.radiantviewer.com).

### Statistical analysis

All the data were analyzed by one-way analysis of variance (ANOVA) or student’s *t*-test with Tukey’s multiple comparisons test using GraphPad Prism 6.0 (CA, USA). The level of significance for all statistical analysis was considered at 0.05.

## Supplementary Information

**Additional file 1: Figure S1.** Fourier-transform infrared (FTIR) spectra of nanocarriers in each step of the catalyst fabrication.** Figure S2.** Energy-dispersive X-ray (EDX) mapping of (A) SPION@MSNs, (B) SPION@MSNs-NH_2_, (C) Au-NPs@DOX and (D) PEG-Au-NPs@DOX. **Figure S3.** Gold NPs were synthesized and characterized by (A) TEM (scale bar is 32 nm), (B) FTIR spectra and (C) UV/vis spectrophotometry. (D) The DLS results showed that gold NPs were around 7.92 nm and (E) zeta potential was around -17.66 mV. **Table S1.** Signal intensity of non-targeted and targeted nanocarriers after 12 and 24 h post injection as revealed by MRI.

## Abbreviations

5-FU: 5-Fluorouracil
APTES: (3-Aminopropyl)triethoxysilane
BET: Brunauer–Emmett–Teller
CHO: Chinese hamster ovary
CTAB: *N*-Cetyltrimethylammonium bromide
DAPI: 4′,6-Diamidino-2-phenylindole
DDS: Drug delivery system
DLS: Dynamic light scattering
DOX: Doxorubicin
EDC: 1-Ethyl-3-(3-dimethylaminopropyl) carbodiimide hydrochloride
EpCAM: Epithelial cell adhesion molecule
EPR: Enhanced permeability and retention
FBS: Fetal bovine serum
FTIR: Fourier-transform infrared
*g*: Gravity
HA: Hyaluronic acid
HR-TEM: High resolution-transmission electron microscopy
MRI: Magnetic resonance imaging
MSN: Mesoporous silica nanoparticle
MTT: 3-(4,5-Dimethylthiazol-2-yl)-2, 5-diphenyltetrazolium bromide
NP: Nanoparticle
NHS: *N*-Hydroxysuccinimide
PEG: Polyethylene glycol
PI: Propidium iodide
RBC: Red blood cell
RPMI: Roswell Park Memorial Institute
SEM: Scanning electron microscopy
SPION: Superparamagnetic iron oxide
TEOS: Tetraethyl orthosilicate
TEM: Transmission electron microscopy
TGA: Thermal gravimetric analysis
TME: Tumor microenvironment
VSM: Vibration sample magnetometer
