# Supplementary material for: Improving anti-cancer drug delivery performance of magnetic mesoporous silica nanocarriers for more efficient colorectal cancer therapy
Source: J Nanobiotechnology. 2021 Oct 12;19:314. doi: 10.1186/s12951-021-01056-3 (PMC8507230; doi:10.1186/s12951-021-01056-3)
Supplement: Supplementary file 1 — Additional file 1: Figure S1. Fourier-transform infrared (FTIR) spectra of nanocarriers in each step of the catalyst fabrication. Figure S2. Energy-dispersive X-ray (EDX) mapping of (A) SPION@MSNs, (B) SPION@MSNs-NH2, (C) Au-NPs@DOX and (D) PEG-Au-NPs@DOX. Figure S3. Gold NPs were synthesized and characterized by (A) TEM (scale bar is 32 nm), (B) FTIR spectra and (C) UV/vis spectrophotometry. (D) The DLS results showed that gold NPs were around 7.92 nm and (E) zeta potential was around -17.66 mV. Table S1. Signal intensity of non-targeted and targeted nanocarriers after 12 and 24 h post injection as revealed by MRI. [file 12951_2021_1056_MOESM1_ESM.docx]

**Additional Materials**

**Improving anti-cancer drug delivery performance of magnetic mesoporous silica nanocarriers for more efficient colorectal cancer therapy**

Sonia Iranpour^a^, Ahmad Reza Bahrami^a,b^, Sirous Nekooei^c^, Amir Sh. Saljooghi^d,e*^, Maryam M. Matin^a,e*^

^a^ Department of Biology, Faculty of Science, Ferdowsi University of Mashhad, Mashhad, Iran

^b^ Industrial Biotechnology Research Group, Institute of Biotechnology, Ferdowsi University of Mashhad, Mashhad, Iran

^c^ Department of Radiology, Faculty of Medicine, Mashhad University of Medical Sciences, Mashhad, Iran

^d^ Department of Chemistry, Faculty of Science, Ferdowsi University of Mashhad, Mashhad, Iran

^e^ Novel Diagnostics and Therapeutics Research Group, Institute of Biotechnology, Ferdowsi University of Mashhad, Mashhad, Iran

* Correspondence: saljooghi@um.ac.ir; matin@um.ac.ir (ORCID: 0000-0002-7949-7712)


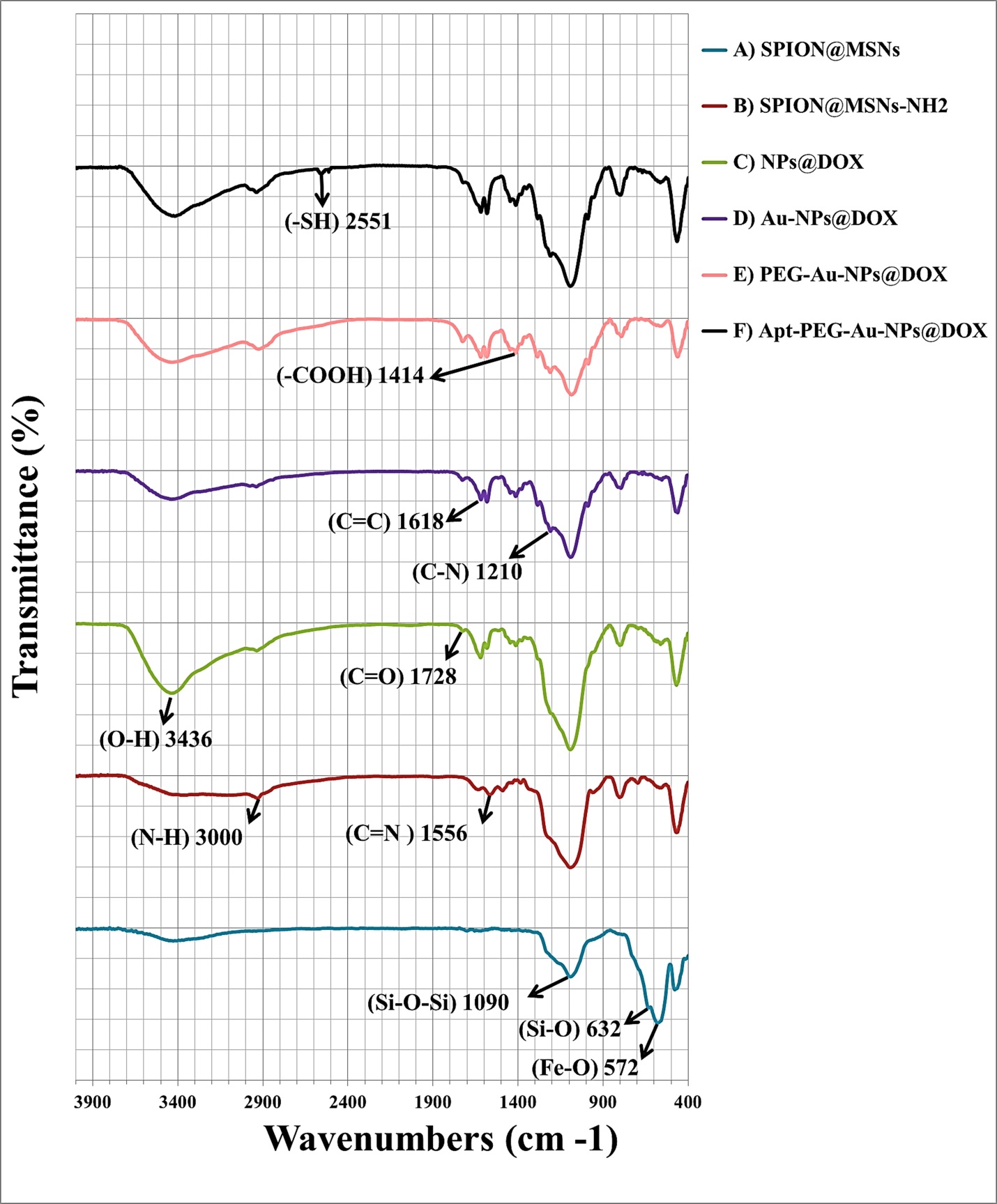


Fig. S1: Fourier transform infrared spectroscopy (FT-IR) spectra of nanocarriers in each step of the catalyst fabrication.

**Abbreviations:** SPION, Superparamagnetic iron oxide nanoparticle; MSN, Mesoporous silica nanoparticle; PEG, Polyethylene glycol; NP, nanoparticle; Apt, Aptamer and DOX, Doxorubicin.


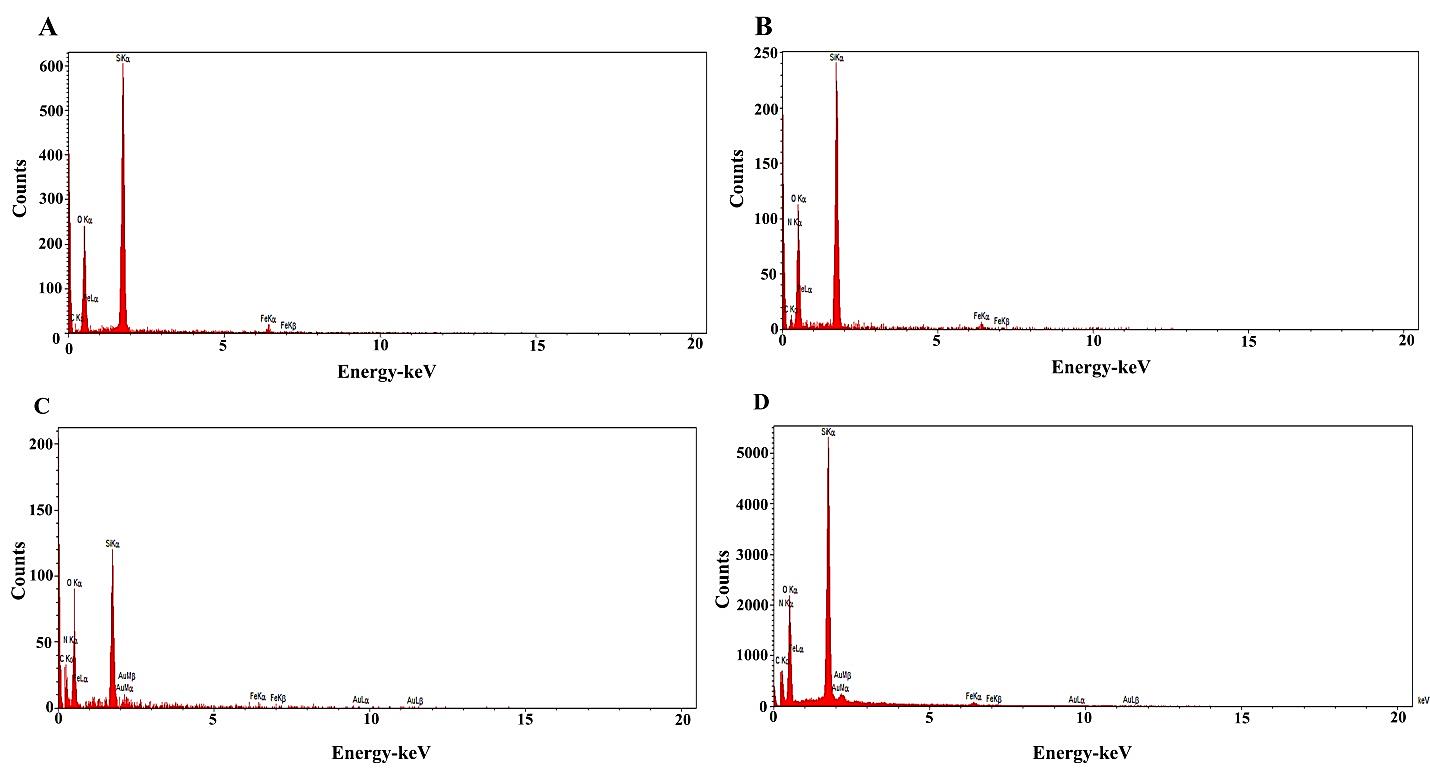


**Fig. S2:** Energy-dispersive X-ray (EDX) mapping of (A) SPION@MSNs, (B) SPION@MSNs-NH_2_, (C) Au-NPs@DOX and (D) PEG-Au-NPs@DOX.

**Abbreviations:** SPION, Superparamagnetic iron oxide nanoparticle; MSN, Mesoporous silica nanoparticle; DOX, Doxorubicin; NP, nanoparticle; PEG, Polyethylene glycol and Apt, Aptamer.


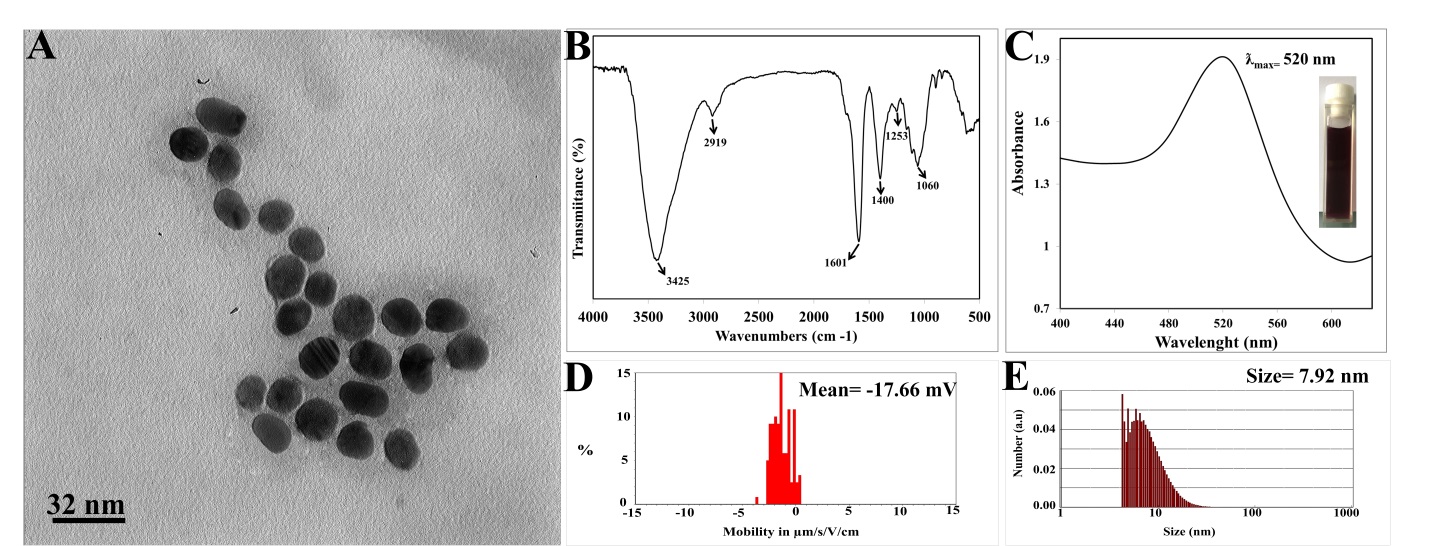


Fig. S3: Gold NPs were synthesized and characterized by (A) TEM (scale bar is 32 nm), (B) FT-IR spectra and (C) UV/vis spectrophotometry. (D) The DLS results showed that gold NPs was around 7.92 nm and (E) zeta potential was around -17.66 mV.

Abbreviations: NP, Nanoparticle; TEM, Transmission electron microscopy; FT-IR, Fourier transform infrared spectroscopy; UV/Vis, Ultraviolet/visible and DLS, Dynamic light scattering.

Table S1: Signal intensity of non-targeted and targeted nanocarriers after 12 and 24 h post injection as revealed by MRI.

| Signal intensity (AU) | 12 h | 24 h |
| --- | --- | --- |
| control | 100 | 100 |
| PEG-Au-NPs@DOX | 78 | 76 |
| Apt-PEG-Au-NPs@DOX | 64 | 62 |

**Abbreviations:** AU, arbitrary unit; PEG, Polyethylene glycol; NP, nanoparticle; DOX, Doxorubicin and Apt, Aptamer.
